# Antiviral Activities of Halogenated Emodin Derivatives against Human Coronavirus NL63

**DOI:** 10.3390/molecules26226825

**Published:** 2021-11-11

**Authors:** Monika Horvat, Martina Avbelj, María Beatriz Durán-Alonso, Mihailo Banjanac, Hrvoje Petković, Jernej Iskra

**Affiliations:** 1 Faculty of Chemistry and Chemical Technology, University of Ljubljana, Večna pot 113, 1000 Ljubljana, Slovenia; monika.horvat@fkkt.uni-lj.si; 2 Biotechnical Faculty, University of Ljubljana, Jamnikarjeva 101, 1000 Ljubljana, Slovenia; martina.avbelj@bf.uni-lj.si; 3 Unit of Excellence, Institute of Biology and Molecular Genetics (IBGM), University of Valladolid-CSIC, 47003 Valladolid, Spain; mariabeatriz.duran@uva.es; 4 Fidelta d.o.o., Prilaz baruna Filipovića 29, 10000 Zagreb, Croatia; mihailo.banjanac@fidelta.eu

**Keywords:** emodin, halogenated emodin, human coronavirus NL63, antiviral activities

## Abstract

The current COVID-19 outbreak has highlighted the need for the development of new vaccines and drugs to combat Severe Acute Respiratory Syndrome-Coronavirus-2 (SARS-CoV-2). Recently, various drugs have been proposed as potentially effective against COVID-19, such as remdesivir, infliximab and imatinib. Natural plants have been used as an alternative source of drugs for thousands of years, and some of them are effective for the treatment of various viral diseases. Emodin (1,3,8-trihydroxy-6-methylanthracene-9,10-dione) is a biologically active anthraquinone with antiviral activity that is found in various plants. We studied the selectivity of electrophilic aromatic substitution reactions on an emodin core (halogenation, nitration and sulfonation), which resulted in a library of emodin derivatives. The main aim of this work was to carry out an initial evaluation of the potential to improve the activity of emodin against human coronavirus NL63 (HCoV-NL63) and also to generate a set of initial SAR guidelines. We have prepared emodin derivatives which displayed significant anti-HCoV-NL63 activity. We observed that halogenation of emodin can improve its antiviral activity. The most active compound in this study was the iodinated emodin analogue **E_3I,** whose anti-HCoV-NL63 activity was comparable to that of remdesivir. Evaluation of the emodin analogues also revealed some unwanted toxicity to Vero cells. Since new synthetic routes are now available that allow modification of the emodin structure, it is reasonable to expect that analogues with significantly improved anti-HCoV-NL63 activity and lowered toxicity may thus be generated.

## 1. Introduction

Severe Acute Respiratory Syndrome-Coronavirus-2 (SARS-CoV-2) is a family of enveloped positive-sense RNA viruses that cause life-threatening respiratory infections and severe pneumonia in humans [1,2]. Coronavirus (CoV) entry into host cells (pulmonary and parabronchial epithelial cells) is mediated by spike protein, which is responsible for binding to receptors ACE-2 and mediating virus–host membrane fusion [3,4,5]. The development of effective antiviral drugs with a broad spectrum of activity has been hampered by viral diversity and the ability of SARS-CoV to mutate rapidly, even during an epidemic. It is therefore very important to develop antiviral drugs that effectively and safely inhibit the spread of SARS-CoV, or at least significantly alleviate the symptoms of SARS-CoV infection. In particular, the development of simple, small compounds that can be produced and administered inexpensively would be of great importance. Recently, several potential repurposed drugs against COVID-19 (SARS-CoV-2 virus) have been found, such as remdesivir, infliximab and imatinib. Remdesivir has potent antiviral activity and has already been approved for urgent use [6,7]. However, to curb the spread of infection, it is important to identify new drug-leads that are more broadly effective against CoV.

Nature is not only a source of emerging mutant viruses, but also a reservoir of natural products that play a crucial role in drug development. Emodin (1,3,8-trihydroxy-6-methylanthracene-9,10-dione), a potent natural bioactive anthraquinone, is found in various plants, lichens and molds, such as *Cassia obtusifolia* and *Cassia occidentalis*, *Rhamnus orbiculatus*, Aloe vera, Japanese knotweed, *Polygonum multiflorum*, *Rheum palmatum*, *Scutellaria baicalensis* and *Rumex chalepensis* [8,9,10,11,12,13]. Emodin is known for its anti-oxidant, anti-ulcerogenic, anti-bacterial, anti-fibrotic, anti-inflammatory, anti-cardiovascular, anti-viral and anti-cancer activities [14,15,16,17,18,19]. It has demonstrated antitumor activity against various cancers such as leukemia, squamous cell carcinoma of human tongue, lung cancer, gallbladder cancer, breast cancer, colon cancer and others [9,14,15,20,21]. Modified emodin compounds have therefore shown relevant pharmacological activity [22,23,24,25,26,27]. Of particular interest and promise are the results obtained with halogenated derivatives of emodin. In 2014, Huang and co-workers found that halogenated emodin derivatives can exert a potent inhibitory activity on bacterial topoisomerase I and DNA gyrase. The best results were obtained with 2,4-diiodoemodin [28]. In 2017, the research group led by Sukhatme and Sun reported the structure–activity relationship (SAR) of emodin and emodin derivatives as ATP citrate lyase (ACL) inhibitors. Halogenated emodin analogues (2-iodoemodin, 2-chloroemodin, 4-chloroemodin and 2,4-dibromoemodin) showed significantly increased activity [29]. Later, Tansakul’s group demonstrated that the hydroxyl and methyl groups were crucial for anti-MRSA (anti-methicillin-resistant Staphylococcus aureus) activity. All compounds containing two halogenated atoms (I, Br or Cl) at positions 2 and 4 were active against MRSA. The best results were obtained in the presence of an iodine atom (2,4-diiodoemodin) [30]. 4-chloroemodin was found to significantly inhibit the growth of gram-positive bacteria, especially that of common drug-resistant MRSA and VRE (vancomycin-resistant enterococci) isolates, through a dual antibacterial mechanism that interacts with the bacterial cell membrane and DNA [31].

In addition, emerging evidence suggests that emodin displays broad spectrum antiviral activities against herpes simplex viruses (HSV-1 and HSV-2) [32,33], hepatitis B virus (HBV) [34,35], Japanese encephalitis virus (JEV) [13], Human cytomegalovirus [36], Influenza A [37], Zika virus [38], Coxsackie B virus [39,40], Poliovirus [41], Cypridine herpesvirus 3 (CyHV-3) [42] and in a number of viral diseases. Through its antiviral activity, emodin can also prevent or reduce SARS-CoV infection [43,44,45,46,47,48]. Hsiang and co-workers reported that emodin can block the interaction of SARS-CoV spike protein with ACE-2 and infectivity of spike protein-pseudotyped retrovirus on Vero E6 cells [49]. In 2011, Schwarz and co-workers showed that emodin can inhibit the 3a ion channel of coronavirus as well as the release of SARS-CoV from infected cells [19]. Singha Roy and Das performed a blind molecular docking analysis of natural anthraquinones against SARS-CoV-2 main protease Mpro. The results suggest that natural emodins may prove to be effective inhibitors of COVID-19 by binding to the catalytic dyad, HIS41 and CYS145, through non-covalent forces near the active site [50].

The aim of the current study was to synthesize a series of emodin analogues and investigate their activities against human coronavirus NL63 (HCoV-NL63) [51]. For this work, we focused on the HCoV-NL63 virus, which causes mild to moderate upper respiratory tract infections in children, severe lower respiratory tract infection, croup and bronchiolitis [52]. HCoV-NL63 and SARS-CoV-2 both belong to the group of common human coronaviruses and they both use the ACE2 receptor to infect cells. HCoV-NL63 is thus a low pathogenic common coronavirus that may be used to study SARS under BSL2 conditions. This is the reason why HCoV-NL63 has been suggested as a suitable surrogate virus for studying SARS-CoV-2 [53].

**Figure 1:**
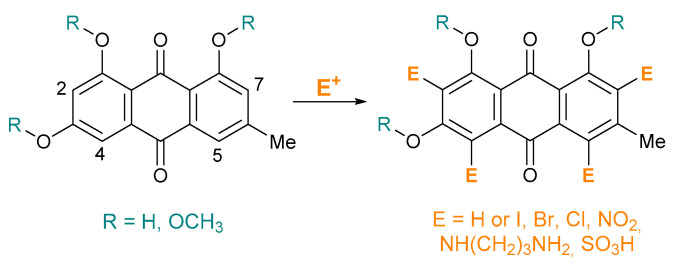
Derivatization of emodin.

Herein, we report the strategies followed for the selective introduction of NO_2_, SO_3_H and halogen atoms into the anthraquinone ring of emodin. Taking into account the potency of halogenated emodins, our work focused on the selective decoration of emodin, containing different halogen atoms and substitution patterns (Figure 1) and the evaluation of their antiviral activity against HCoV-NL63. Our results demonstrate that the presence of different functional groups in the emodin scaffold has a significant impact on their anti-HCoV-NL63 activity.

## 2. Results and Discussion

### 2.1. Synthesis of Emodin Derivatives

The natural product emodin **E_H** provides an entry point for the introduction of various functional groups on aromatic rings by electrophilic aromatic substitution. Our aim was to create a library of emodin derivatives by modifying the aromatic ring through halogenation, nitration, amination and sulfonation.

First, we investigated the selective halogenation of emodin **E_H** by classical reagents (*N*-chlorosuccinimide—NCS, *N*-bromosuccinimide—NBS, *N*-iodoosuccinimide—NIS) and by an alternative method—oxidative halogenation with hydrogen peroxide as oxidant. 2-Iodoemodin **E_I** was prepared according to the published methods [28] using I_2_ and NaHCO_3_ in 73% yield (Table 1, entry 1) and in the presence of NIS reagent in 84% yield (Table 1, entry 6). Oxidative iodination with I_2_ and 30% hydrogen peroxide in 2-MeTHF also allowed the selective synthesis of the same product with a better yield of 93% (Table 1, entry 16). For the oxidative iodination, 2 equivalents of iodine and 2.5 equivalents of hydrogen peroxide were used. While conducting the experimental work, it was observed that the reaction does not proceed in the absence of the oxidant H_2_O_2_ (entry 11) and that a higher amount of iodine is required for the quantitative conversion to **E_I** (entries 12–15). We also found that the amount of hydrogen peroxide has a minimal effect on the increase in conversion. A larger number of iodine substituents could not be introduced into the aromatic core of emodin using oxidative iodination. To introduce additional iodine atoms, I_2_/NaHCO_3_ or NIS had to be used. Nevertheless, the diiodinated emodin **E_2I** could not be selectively prepared, regardless of the amount of iodine or NIS used. 2,4,7-Triiodoemodin **E_3I** was selectively formed by applying the classical method (I_2_, NaHCO_3_) [28] in 81% yield or using NIS reagent in 79% yield as a brown-orange powder (Table 1, entries 3 and 10). We also attempted to synthesize the fully iodinated emodin **E_4I**, but this compound could not be prepared regardless of the reaction conditions. We tried increasing the temperature and adding the activator H_2_SO_4_ to the reagent NIS. In all cases, the triiodinated product appeared selectively. The positions of iodine on the aromatic rings in compounds **E_I** and **E_3I** were confirmed by 2D NMR spectroscopy (Figures S6–S13, Supplementary Material).

Next, bromination was carried out using NBS and by oxidative halogenation using H_2_O_2_/HBr. Neither of these methods could be effective for selective synthesis of monobromo-derivative **E_Br**, as shown by the results in Table 2. Although a reaction with 1 equivalent of NBS was carried out in an ice bath, both **E_Br** and **E_2Br** were formed simultaneously (Table 2, entries 1 and 2). The same results were observed for oxidative bromination with HBr and H_2_O_2_. Regardless of the amounts of HBr and hydrogen peroxide used, a mixture of the two products was always formed. Using 2D NMR spectroscopy, we found that in the case of **E_Br**, bromine binds to site 2 in emodin, either when the NBS reagent or HBr/H_2_O_2_ is used.

**E_2Br** was selectively prepared according to a published procedure [29] with a slight modification consisting of treating emodin **E_H** with NBS in THF at 0 °C (88% yield). Due to the high reactivity of emodin, the bromination required a relatively short reaction time (30 min) and a low temperature (0 °C). The same product was obtained by the oxidative halogenation method (HBr, H_2_O_2_) in 2,2,2 trifluoroethanol (TFE) in 91% yield (Table 2, entry 14). Unfortunately, the oxidative method, although more environmentally friendly, did not allow the introduction of more bromine atoms. Despite the higher amount of HBr and the use of the activating solvent TFE, the reaction stopped at the dibrominated product. The use of an NBS reagent allowed a greater number of bromine atoms to be introduced, but we encountered problems with the selectivity in the preparation of **E_3Br**. Regardless of the temperature at which the reaction was carried out and the amount of reagent used, **E_2Br** and/or **E_4Br** were also formed (Table 2, entries 4–6). Selectively, we prepared the orange-colored product 2,4,6,8-tetrabromo-1,3,5-trihydroxy-7-methylanthracene-9,10-dione **E_4Br** (Table 2, entry 7).

Chlorination of emodin gave similar results to bromination. The monosubstituted product could not be prepared selectively with the NCS reagent or oxidatively with HCl/H_2_O_2_, since the dichloro derivative **E_2Cl** was also formed. It was observed that the regioselectivity of chlorination to **E_Cl** depends on the method used—HCl/H_2_O_2_ or NCS—as determined by conducting 1D and 2D NMR spectroscopy on the crude reaction mixture consisting of **E_H**, **E Cl** or/and **E_2Cl**. The results showed that when NCS was used, the Cl was bound at position 2 (**E_Cl-2**), whereas when HCl/H_2_O_2_ was used, it was bound at position 4 (**E_Cl-4**) (Figures S1–S3). Good yields of the disubstituted emodin 2,4-dichloroemodin **E_2Cl** were obtained selectively with NCS [30] using H_2_SO_4_ as catalyst in a 3-h process at room temperature (Table 3, entry 3). When emodin was treated with 6 equivalents of NCS in the presence of H_2_SO_4_ at a reflux temperature, no formation of three- or four-chloroemodin occurred. Only two chlorine atoms could be introduced into emodin. Oxidative chlorination was proved to be an unsuitable procedure for the selective synthesis of chlorine products. Despite the combination of different ratios of HCl and H_2_O_2_, emodin was always converted into a mixture of different products that could not be separated (Table 3, entries 5–9).

Next, we investigated the selective nitration of **E_H**. We attempted to selectively prepare nitroemodin with one, two or three NO_2_ functional groups, but none of the conditions tested led to successful results. The results showed that despite the use of small amounts of nitric and sulfuric acids, a mixture of various nitrated products **E_X** formed that could not be separated (Table 4, entries 1–7). It was concluded that the nitration reaction is not selective towards a particular product despite the absence of H_2_SO_4_. We prepared the tetranitroemodin **E_4NO_2_** using 6.2 equiv. HNO_3_ and 10 equiv. H_2_SO_4_ in 91% yield (Table 4, entry 8), reducing excess reagents by an order of magnitude according to a published method [24] (Table 4, entry 9).

**Figure 2:**
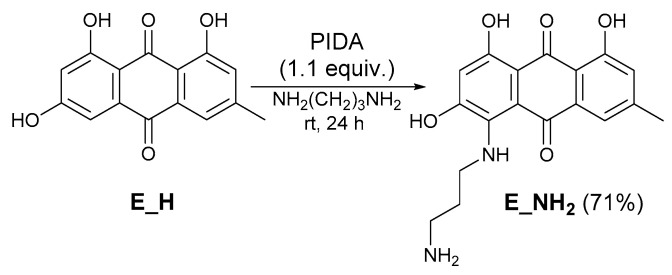
Synthesis of amino emodin **E_NH_2_**.

In addition, we also prepared amino-substituted emodin **E_NH_2_** by the method described in an earlier publication (Figure 2) [24].

**Figure 3:**
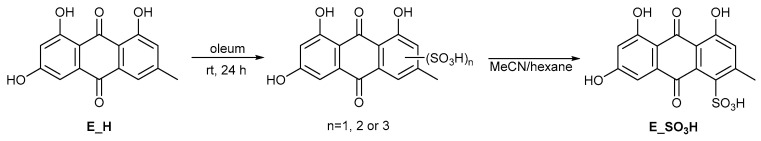
Synthesis of **E_SO_3_H**.

Sulfonation was carried out with oleum at room temperature. After 24 h, the reaction mixture was analyzed by NMR; three different products were observed. According to the NMR spectra obtained from the crude reaction mixture, trisulfonated, disulfonated and monosulfonated emodin were formed. However, the composition of the mixture changed with time and reverse sulfonation occurred. Hence, following the work-up procedure, the crude reaction mixture was stirred in MeCN/hexane and after 3 h the product was completely converted to a stable monosubstituted **E_SO_3_H**, which was isolated as a brown solid in 76% yield (Figure 3).

1,3,8-Trimethoxy-6-methyl-9,10-anthraquinone **E_OCH_3_** was obtained in good yield (81%) by applying a previously described reaction procedure (Me_2_SO_4_ and base K_2_CO_3_) [30]. Brominated trimethoxyemodin **E_Br_OCH_3_** was synthesized from tetrabrominated emodin **E_4Br** by the same method. After purification, the product **E_4Br_OCH_3_** was obtained in 87% yield.

### 2.2. Antiviral Activity of Emodin Analogues

**Figure 4:**
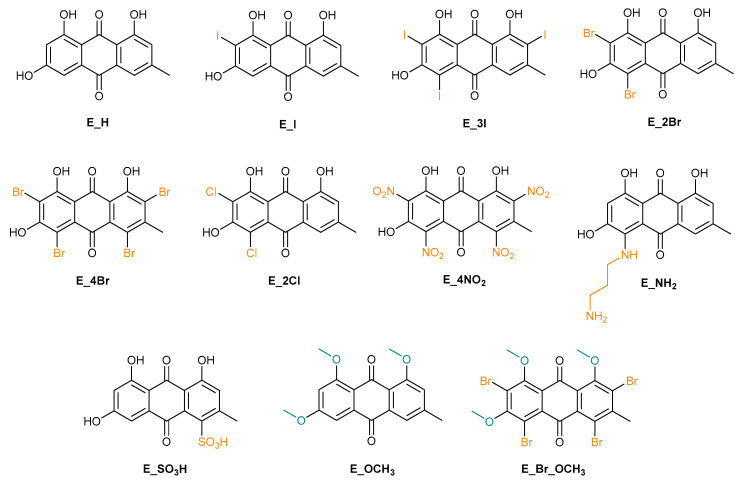
Emodin and emodin derivatives prepared in the course of this study.

The primary objective of this study was to determine the potential of a library of 11 emodin analogues (Figure 4) to combat the cytopathic effects of HCoV-NL63. The non-transformed primate kidney Vero cell line was used for this work. An additional series of studies was performed to evaluate the effects of each compound on the viability of Vero cells.

#### 2.2.1. Evaluation of the Viability of Vero Cells in the Presence of Emodin and Emodin Analogues

**Figure 5:**
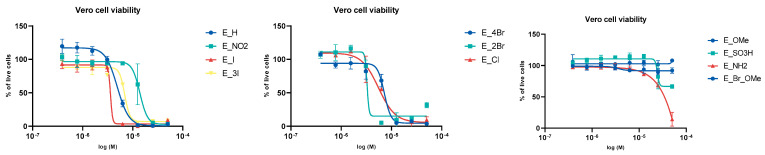
IC50 curves reflecting the effect of the tested compounds on Vero cell viability.

Published reports indicate that emodin displays a broad spectrum of activities, including cytotoxicity [14]. Therefore, in addition to evaluating the activity of emodin and the emodin analogues against HCoV-NL63, it was important to test the potential cytotoxicity of these compounds. Viability assays were performed using Vero cells; IC50 curves for each compound and other additional data are shown in Figure 5 and in Table S1 (raw data in Table S4).

Interestingly, only **E_I** showed higher toxicity to Vero cells compared to emodin (**E_H**). **E_4Br**, **E_Cl** and **E_OMe** were less toxic than emodin, while virtually no toxicity was observed with **E_SO_3_H**, **E_NH_2_** and **E_Br_OMe**. However, it is important to consider that although all emodin analogues were soluble in DMSO at 50 mM concentrations, some analogues exhibited a tendency to precipitate when added to the cell culture medium; compounds containing methoxy groups were the most difficult to dissolve in DMSO and were therefore probably the least soluble in aqueous solutions. It is therefore possible that these compounds gave lower cytotoxicity indexes due to their poor solubility in the cell culture medium.

#### 2.2.2. Anti-Viral Activity of Emodin and Emodin Derivatives

**Figure 6:**
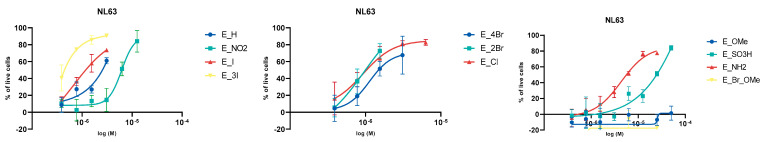
IC_50_ curves representing the anti-HCoV-NL63 effects of emodin and emodin derivatives.

In another series of studies, we evaluated the potential of emodin and emodin derivatives to protect Vero cells against the cytopathic effects induced by HCoV-NL63. Dead cell protease activity was used as a measure of impaired cell viability, as described in the Methods section; chloroquine and remdesivir were used as controls. The results obtained are presented in Figure 6 and Table 5. Additional data can be found in Supplementary Materials (Table S2; raw data in Table S5). Three compounds, **E_OMe**, **E_SO_3_H** and **E_Br_OMe**, did not exhibit significant anti-viral activity; as described above, this could be due to their lower solubility in aqueous solutions. Emodin and the emodin analogues **E_4NO_2_** and **E_I** impaired Vero cell viability and had anti-viral effects at very similar concentrations. Five other compounds, **E_3I**, **E_4Br**, **E_2Br**, **E_2Cl** and **E_NH_2_**, showed anti-viral activity at concentrations lower than those at which they impaired Vero cell viability; among these, **E_NH_2_** exhibited the least toxicity to Vero cells. On the other hand, E_3I was the compound that demonstrated the strongest anti-HCoV-NL63 activity, along with the largest difference (about 10-fold) between the IC_50_ value for anti-HCoV-NL63 activity and that for cytotoxicity, thus providing the largest therapeutic window. Nevertheless, the IC_50_ value for cytotoxicity of **E_3I** was still considerably high under the applied experimental conditions, namely identical to that of **E_H** (Table S1).

As mentioned earlier, chloroquine and remdesivir were used as controls, since these drugs are being considered as potentially effective against COVID-19. Interestingly, the anti-HCoV-NL63 activity of some of the emodin analogues was much higher than that of chloroquine. On the other hand, the emodin analogues **E_3I** and **E_2Br** displayed anti-HCoV-NL63 activity comparable to that of remdesivir; all the data obtained on the activities of chloroquine and remdesivir are presented in Supplementary Materials (Table S3 and Figure S5; raw data in Tables S6 and S7).

## 3. Materials and Methods

Emodin was purchased from Fluorochem Ltd. (Glossop, UK). All other reagents and solvents were of reagent-grade quality and were obtained from commercial suppliers Honeywell (Seelze, Germany) and Sigma-Aldrich (Taufkirchen, Germany). TLC was performed on Merck-60-F_254_ plates (Merck, Darmstadt, Germany) using mixtures of EtOAc:EtOH (10:1), CH_2_Cl_2_:EtOH (100:1) or EtOAc:MeOH (20:1). Crude emodin preparations were purified by column chromatography on silica gel (63–200 µm, 70–230 mesh ASTM; Honeywell, Seelze, Germany). The isolated compounds were characterized by ^1^H, ^13^C NMR spectra, HRMS and IR analysis. ^1^H and ^13^C NMR spectra were recorded on Bruker Avance III 500 instruments (Bruker, Billerica, MA, USA). IR spectra were recorded on Bruker Alpha II FTIR Instrument (Bruker, Billerica, MA, USA). HR-MS were recorded on LC MS system Agilent 6224 Accurate Mass TOF LC/MS (Agilent Technologies, Santa Clara, CA, USA).

### 3.1. Compound Synthesis and Structure Confirmation

1,3,8-trihydroxy-2-iodo-6-methylanthracene-9,10-dione (E_I) [28]. Iodine (254 mg, 1.0 mmol) and 30% H_2_O_2_ (255 µL, 2.5 mmol) were added to a stirred solution of emodin (135 mg, 0.5 mmol) in 2-MeTHF (5 mL). The reaction mixture was stirred at room temperature for 24 h. The reaction was monitored by TLC (CH_2_Cl_2_:EtOH = 100:1). After the reaction was complete, the reaction mixture was washed with NaHSO_3_ and extracted with dichloromethane (3 × 30 mL). The organic layer was washed with water (1 × 30 mL), dried over anhydrous Na_2_SO_4_ and evaporated under a vacuum. The crude reaction product was washed with hexane (5 mL) and acetonitrile (5 mL) to remove soluble impurities. The product was dried in vacuum to provide the product (184 mg, 93%) as an orange solid. ^1^H NMR (500 MHz, DMSO-*d*_6_, 25 °C): *δ* = 13.07 (s, 1H, OH), 12.26 (s, 1H, OH), 11.83 (s, 1H, OH), 7.47 (s, 1H, ArH), 7.21 (s, 1H, ArH), 7.16 (s, 1H, ArH), 2.41 (s, 3H, CH_3_) ppm. ^13^C NMR (126 MHz, DMSO-*d*_6_, 25 °C): *δ* = 189.4, 181.2, 165.2, 163.5, 161.4, 148.6, 134.2, 132.7, 124.3, 120.6, 113.1, 108.5, 106.8, 83.2, 21.6 ppm. IR: 3339, 1665, 1615, 1473, 1379, 1263, 1168, 949 cm^−1^. HRMS (ESI^−^): *m/z* calcd for C_15_H_9_IO_5_ 394.9422 [M-H]^−^, found: 394.9428 [M-H]^−^.

1,3,8-trihydroxy-2,4,7-triiodo-6-methylanthracene-9,10-dione (E_3I) [28]. Iodine (1.3 g, 5.0 mmol) was added at a temperature of 0 °C to a stirred solution of emodin (135 mg, 0.5 mmol) in THF (13 mL) and water (13 mL). NaHCO_3_ (3.5 g, 42.0 mmol) was then added in a stepwise manner. The reaction mixture was stirred for 24 h at room temperature. The reaction was monitored by TLC (CH_2_Cl_2_:EtOH = 100:1). After completion of the reaction, the mixture was extracted with dichloromethane (3 × 30 mL). The organic layer was washed with water (1 × 30 mL), dried over anhydrous Na_2_SO_4_ and evaporated under vacuum. The crude reaction product was washed with hexane (5 mL) and acetonitrile (3 × 5 mL) to remove soluble impurities. The product was dried in vacuum to provide the product (262 mg, 81%) as an orange–brown solid. ^1^H NMR (500 MHz, DMSO-*d*_6_, 25 °C): *δ* = 13.61 (s, 1H, OH), 12.60 (s, 1H, OH), 7.47 (s, 1H, ArH), 2.48 (s, 3H, CH_3_) ppm. ^13^C NMR (126 MHz, DMSO-*d*_6_, 25 °C): *δ* = 187.2, 180.2, 165.1, 163.8, 159.5, 151.8, 133.3, 132.0, 120.4, 111.8, 110.0, 101.3, 84.1, 82.5, 29.3 ppm. IR: 3359, 1614, 1377, 1236, 1111, 1043 cm^−1^. HRMS (ESI^−^): *m/z* calcd for C_15_H_7_I_3_O_5_ 646.7355 [M-H]^−^, found: 646.7380 [M-H]^−^.

2,4-dibromo-1,3,8-trihydroxy-6-methylanthracene-9,10-dione (E_2Br) [28]. HBr (48%, 2.0 mmol) and 30% H_2_O_2_ (255 µL, 2.5 mmol) were added to a stirred solution of emodin (135 mg, 0.5 mmol) in TFE (5 mL). The reaction mixture was stirred at room temperature for 24 h. The reaction was monitored by TLC (CH_2_Cl_2_:EtOH = 100:1). After completion of the reaction, the mixture was washed with NaHSO_3_ and extracted with CH_2_Cl_2_ (3 × 30 mL). The organic layer was washed with water (1 × 30 mL), dried over anhydrous Na_2_SO_4_ and evaporated under a vacuum. The crude reaction product was washed with hexane (5 mL) and acetonitrile (5 mL) to remove soluble impurities. The product was dried in a vacuum to provide the product (194.7 mg, 91%) as an orange solid. ^1^H NMR (500 MHz, DMSO-*d_6_*, 25 °C): *δ* = 13. 89 (s, 1H, OH), 12.10 (s, 1H, OH), 7.42 (d, *J* = 0.6 Hz, 1H, ArH), 7.09 (d, *J* = 0.6 Hz, 1H, ArH), 2.40 (s, 3H, CH_3_) ppm. ^13^C NMR (126 MHz, DMSO-*d*_6_, 25 °C): *δ* = 188.8, 181.2, 161.1, 160.7, 149.2, 134.0, 130.5, 123.9, 121.1, 113.2, 110.6, 106.6, 105.7, 67.5, 22.1 ppm. IR: 3346, 1664, 1376, 1289, 1250, 1213, 1114 cm^−1^. HRMS (ESI^−^): *m/z* calcd for C_15_H_8_Br_2_O_5_ 424.8666 [M-H]^−^, found: 424.8667 [M-H]^−^.

2,4,5,7-tetrabromo-1,3,8-trihydroxy-6-methylanthracene-9,10-dione (E_4Br) [54]. *N*-Bromosuccinimide (890 mg, 5.0 mmol) was added to a solution of emodin (270 mg, 1.0 mmol) in THF (5 mL) and stirred for 24 h at room temperature. After completion of the reaction, the mixture was extracted with ethyl acetate (3 × 30 mL). The organic layer was dried over anhydrous Na_2_SO_4_ and the solvent was evaporated under a vacuum. The crude reaction product was washed with hexane (5 mL) and acetonitrile (3 × 5 mL) to remove soluble impurities. The product was dried in a vacuum to provide the product (486 mg, 83%) as an orange solid. ^1^H NMR (500 MHz, DMSO-*d*_6_, 25 °C): *δ* = 2.72 (s, 3H, CH_3_) ppm. ^13^C NMR (126 MHz, DMSO-*d*_6_, 25 °C): *δ* = 185.4, 183.0, 162.5, 159.5, 156.3, 147.3, 133.5, 132.5, 119.7, 115.6, 113.4, 107.9, 106.8, 103.8, 25.6 ppm. IR: 3302, 1679, 1621, 1566, 1379, 1223, 1161, 1129, 1049, 805, 760. IR: 3404, 1666, 1617, 1368, 1315, 1231, 1205, 1123, 1046 cm^−1^. HRMS (ESI^−^): *m/z* calcd for C_15_H_6_Br_4_O_5_ 580.6876 [M-H]^−^, found: 580.6886 [M-H]^−^.

2,4-dichloro-1,3,8-trihydroxy-6-methylanthracene-9,10-dione (E_2Cl) [30]. *N*-Chlorosuccinimide (333.8 mg, 2.5 mmol) and conc. H_2_SO_4_ (0.5 mL) were added to a solution of emodin (270 mg, 1.0 mmol) in THF (5 mL) and stirred for 3 h at room temperature. After the reaction was complete, the mixture was extracted with ethyl acetate (3 × 30 mL). The organic layer was dried over anhydrous Na_2_SO_4_ and evaporated under vacuum. The crude reaction product was washed with hexane (5 mL) and acetonitrile (3 × 5 mL) to remove soluble impurities. The product was dried in vacuum to provide the product (274.7 mg, 81%) as a yellow solid. ^1^H NMR (500 MHz, DMSO-*d*_6_, 25 °C): *δ* = 13.57 (s, 1H, OH), 11.70 (s, 1H, OH), 7.47 (s, 1H, ArH), 7.16 (s, 1H, ArH), 2.42 (s, 3H, CH_3_) ppm. ^13^C NMR (126 MHz, DMSO-*d*_6_, 25 °C): δ = 185.0, 182.0, 165.6, 160.7, 160.2, 147.2, 133.9, 127.5, 123.4, 122.3, 120.3, 113.5, 112.6, 104.2, 22.0 ppm. IR: 3312, 1622, 1536, 1379, 1199, 1162, 1100 cm^−1^. HRMS (ESI^−^): *m/z* calcd for C_15_H_8_Cl_2_O_5_ 336.9676 [M-H]^−^, found: 336.9686 [M-H]^−^.

1,3,8-trihydroxy-6-methyl-2,4,5,7-tetranitroanthracene-9,10-dione (E_4NO_2_) [24]. HNO_3_ (64%, 0.5 mL) was added dropwise over 10 min onto a mixture of emodin (270 mg, 1.0 mmol) and concentrated H_2_SO_4_ (96%, 5 mL) at 0 °C. The reaction mixture was stirred at 0 °C for 30 min. The mixture was poured onto ice and extracted with ethyl acetate (3 × 30 mL). The organic layer was dried over anhydrous Na_2_SO_4_ and the solvent was evaporated under vacuum. The crude reaction was purified by column chromatography using an ethyl acetate/ethanol (10/1) mobile phase. The solvent was evaporated in vacuum to provide the product (408 mg, 91%) as an orange–red solid. ^1^H NMR (500 MHz, DMSO-*d*_6_, 25 °C): *δ* = 2.21 (s, 3H, CH_3_) ppm. ^13^C NMR (126 MHz, DMSO-*d*_6_, 25 °C): *δ* = 183.8, 177.3, 161.0, 156.6, 151.9, 143.2, 140.4, 139.9, 134.8, 128.8, 124.3, 122.4, 117.0, 96.7, 12.3 ppm. IR: 3351, 1638, 1538, 1369, 1170 cm^−1^. HRMS (ESI^+^): *m/z* calcd for C_15_H_6_N_4_O_13_ 451.0004 [M+H]^+^, found: 451.0002 [M+H]^+^.

4-((3-aminopropyl)amino)-1,3,8-trihydroxy-6-methylanthracene-9,10-dione (E_NH_2_) [24]. (Diacetoxyiodo)benzene (354 mg, 1.1 mmol) was added to a solution of emodin (270 mg, 1.0 mmol) in 1,3-diaminopropane (40 mL). The reaction mixture was stirred for 24 h at room temperature. After the reaction was complete, the mixture was poured into cold water (200 mL), 10 M HCl (60 mL), neutralized with saturated NaHCO_3_ (200 mL), and extracted with ethyl acetate (3 × 75 mL). The organic layer was dried over anhydrous Na_2_SO_4_ and evaporated under vacuum. The crude reaction product was purified by column chromatography using ethyl acetate/methanol (20/1) mobile phase. The solvent was evaporated in vacuum to provide the product (243 mg, 71%) as a violet solid. ^1^H NMR (500 MHz, DMSO-*d*_6_, 25 °C): *δ* = 14.92 (s, 1H, OH), 13.61 (s, 1H, OH), 12.34 (s, 1H, OH), 7.50 (s, 1H, ArH), 6.81 (s, 1H, ArH), 5.53 (s, 1H, ArH), 4.17 (q, *J* = 6.4 Hz, 2H, CH_2_), 2.89 (t, *J* = 6.9 Hz, 2H, CH_2_), 2.36 (s, 3H, CH_3_), 1.93–1.87 (m, 2H, CH_2_) ppm. ^13^C NMR (126 MHz, DMSO-*d*_6_, 25 °C): *δ* = 179.2, 176.6, 173.1, 168.4, 160.9, 149.7, 142.7, 135.2, 119.5, 117.5, 116.5, 107.4, 105.6, 100.4, 41.3, 36.9, 29.6, 22.1 ppm. IR: 3195, 1738, 1576, 1502, 1401, 1355 cm^−1^. HRMS: *m/z* calcd for C_18_H_18_N_2_O_5_ 343.1288 [M+H]^+^, found: 343.1290 [M+H]^+^.

1,3,8-trihydroxy-6-methyl-9,10-dioxo-9,10-dihydroanthracene-5-sulfonic acid (E_SO_3_H). Oleum (8 mL) was added dropwise onto emodin (270 mg, 1.0 mmol) for over 15 min at room temperature. The reaction mixture was stirred for 24 h at room temperature. The reaction mixture was poured onto ice and extracted with ethyl acetate (3 × 30 mL). The organic layer was dried over anhydrous Na_2_SO_4_ and the solvent was evaporated under a vacuum. The crude reaction product was stirred in MeCN and hexane for 3 h and was then purified by column chromatography using ethyl acetate/ethanol (10/1) mobile phase. The solvent was evaporated in vacuum to provide the product (266 mg, 76%) as a brown solid. ^1^H NMR (500 MHz, DMSO-*d*_6_, 25 °C): *δ* = 11.40 (s, 1H, OH), 11.16 (s, 1H, OH), 7.35 (d, *J* = 1.9 Hz, 1H, ArH), 7.14 (d, *J* = 1.9 Hz, 1H, ArH), 6.75 (s, 1H, ArH), 2.38 (s, 3H, CH_3_) ppm. ^13^C NMR (126 MHz, DMSO-*d*_6_, 25 °C): *δ* = 158.2, 151.1, 146.8, 139.7, 134.7, 133.5, 126.7, 121.4, 112.1, 110.6, 109.4, 108.9, 105.0, 96.7, 14.6 ppm. IR: 3451, 1738, 1600, 1426, 1367, 1202, 1094 cm^−1^. HRMS (ESI^−^): *m/z* calcd for C_15_H_10_O_8_S 349.0024 [M-H]^−^, found: 349.0032 [M-H]^−^.

1,3,8-trimethoxy-6-methylanthracene-9,10-dione (E_OCH_3_) [30]. Potassium carbonate (415 mg, 3.0 mmol) was added to a solution of emodin (100 mg, 0.37 mmol) in acetone (7 mL). Then dimethyl sulfate (285 µL, 3.0 mmol) was added slowly and the reaction mixture was stirred at reflux for 24 h. The reaction mixture was allowed to cool to room temperature. After cooling to room temperature, the solvent was evaporated. Then water (5 mL) and acetone (5 mL) were added to the reaction mixture under stirring for 15 min. The product was filtrated off, washed with water, and dried in vacuum to provide the product (94 mg, 81%) as a yellow–white solid. ^1^H NMR (500 MHz, Chloroform-*d*, 25 °C): δ = 7.64 (s, 1H, ArH), 7.32 (s, 1H, ArH), 7.09 (s, 1H, ArH), 6.76 (s, 1H, ArH), 3.98 (s, 3H, OCH_3_), 3.96 (s, 3H, OCH_3_), 3.95 (s, 3H, OCH_3_), 2.47 (s, 3H, CH_3_) ppm. ^13^C NMR (126 MHz, Chloroform-*d*, 25 °C): δ = 184.4, 181.8, 163.7, 161.7, 159.8, 144.6, 136.4, 134.4, 121.5, 119.6, 119.0, 118.4, 105.3, 101.9, 56.5, 56.5, 55.9, 22.1 ppm. IR: 1657, 1599, 1322, 1241, 1021, 946, 910 cm^−1^. HRMS (ESI^+^): *m/z* calcd for C_18_H_16_O_5_ 313.1071 [M+H]^+^, found: 313.1077 [M+H]^+^.

2,4,5,7-tetrabromo-1,3,8-trimethoxy-6-methylanthracene-9,10-dione (E_Br_OCH_3_). Potassium carbonate (415 mg, 3.0 mmol) was added to a solution of brominated emodin **5** (216 mg, 0.37 mmol) in acetone (7 mL). Then dimethyl sulfate (285 µL, 3.0 mmol) was added slowly and the reaction mixture was heated to reflux for 24 h. The reaction mixture was allowed to cool to room temperature. After cooling to room temperature, the solvent was evaporated. Then water (5 mL) and acetone (5 mL) were added to the reaction mixture with stirring for 15 min. The product was filtrated off, washed with water and dried in a vacuum to provide the product (202 mg, 87 %) as a light pink solid. ^1^H NMR (500 MHz, Chloroform-*d*, 25 °C): *δ* = 4.02 (s, 3H, OCH_3_), 4.01 (s, 3H, OCH_3_), 3.98 (s, 3H, OCH_3_), 2.78 (s, 3H, CH_3_) ppm. ^13^C NMR (126 MHz, Chloroform-*d*, 25 °C): *δ* = 184.1, 179.5, 160.1, 156.9, 155.1, 147.0, 135.8, 135.0, 128.7, 128.0, 126.7, 121.7, 117.7, 112.2, 63.2, 63.1, 61.1, 25.9 ppm. IR: 2153, 2036, 1695, 1367, 1322, 1216, 991 cm^−1^. HRMS: *m/z* calcd for C_18_H_12_Br_4_O_5_ 624.7491 [M+H]^+^, found: 624.7487 [M+H]^+^.

### 3.2. Evaluation of Antiviral Activity

Compound preparation. For testing purposes, all compounds were dissolved in DMSO to a final concentration of 50 mM. With these stock solutions, mother plates were prepared in DMSO and generated stocks for testing 8 point–dose responses. 1:1 dilutions were prepared, starting from the original 50 mM stock. Namely, 30 µL of stock solution was added to the first column in the plate. 15 µL of DMSO was added to all the other wells; stocks to test successive doses were produced by transferring 15 µL of compound solution from the preceding column to the next. Each mother plate held the test solutions corresponding to two compounds. Chloroquine and Remdesivir were prepared in three-fold dilutions, chloroquine starting at a 30 µM concentration and Remdesivir at 10 µM.

Cell culturing and viral infection. Vero cells (Cercopithecus aethiops lung epithelial cells, ATCC, CRL-81), were seeded in EMEM medium containing 10% FBS in inner wells of 96-well white plates, at a density of 20000 cells/well. The following day, the plating medium was removed and replaced with 100 µL of fresh EMEM medium containing 2% FBS; a 100 nL solution of each compound was then also added to the wells using a Mosquito pipetting device (TTPlabtech, Cambridge, England). Cells were kept in this medium for 4 days at 33 °C in 5% CO_2_ before evaluating the cytotoxicity of each compound.

When conducting experiments to evaluate the anti-viral activity of the emodin compounds, the culture medium was changed at 24 h post-plating to 50 µL of fresh EMEM medium containing 2% FBS; 100 nL solution of each test compound was also added to the wells, as described above and an additional 50 µL of the same medium containing a 1:5 dilution of a HCoV-NL63 viral stock was added (Human Coronavirus, Strain NL63, FR-304, IRR (International Reagent Resource). Cultures were maintained in this medium for 4 days at 33 °C, in 5% CO_2_; the anti-viral activity of each compound was then measured.

### 3.3. Evaluation of Cytotoxic and Anti-Viral Activities

In order to evaluate any potential cytotoxicity of each of the test compounds, 50 µL/well of Cell Titer-Glo Luminescent reagent was added to each culture following a 4 day-incubation in the presence of these compounds. Luminescence was measured after 5 min of incubation using a Spectra Max i3 instrument (Molecular Devices, San Jose, CA, USA); the obtained values were considered proportional to cellular ATP content. Cytotoxic activity of the tested compounds on Vero cells was expressed as the percentage of live cells compared to that in control cultures that had not been exposed to any compound and had been assigned live cell rates of 100%.

Assays to evaluate anti-viral activity were carried out at 4 days post-infection by adding 50 µL/well of Cyto Tox-Fluor reagent to cultures priorly infected with HCoV-NL63 virus. Plates were incubated for 1 h and dead-cell protease activity was assayed by measuring the fluorescent product that had formed (480/520), using a Spectra Max i3 instrument. Anti-viral activity of each compound was presented as the percentage of live cells compared to that in control Vero cultures that had been infected with the HCoV-NL63 virus but had not been exposed to any of the test compounds; these latter cultures were assigned live cell rates of 100%. All IC_50_ values were calculated using GraphPad Prism 8 software (GraphPad Software, LLC, San Diego, CA, USA).

Assays were considered valid when the signal to background ratio (signal from cells infected with the virus versus signal from uninfected cells) was higher than 2 and Z’ was higher than 0.35.

## 4. Conclusions

Emodin is a natural anthraquinone commonly found in plants. Numerous studies (reviewed by Dong and coworkers [14]) show that emodin has a wide spectrum of pharmacological properties. Among these, it is now clear that it also has anti-viral activity. However, emodin can also cause toxicity, such as hepatotoxicity and nephrotoxicity. In accordance with these data, our study demonstrated the toxicity of emodin to Vero cells, a kidney cell line. The main objective of this work was to apply a series of synthetic approaches to the preparation of emodin analogues and to subsequently conduct structure-activity relation (SAR) studies on them, which could shed light on how to improve the anti-HCoV-NL63 activity and to reduce the cytotoxicity of these compounds. Our work, in agreement with the published literature reporting improved activity of halogenated emodin derivatives, showed that halogenation of emodin resulted in enhanced anti-HCoV-NL63 activity; however, cytotoxicity was still relatively high. On the other hand, the introduction of charged groups, such as SO_3_H and NH_2_, resulted in a significant decrease in undesirable cytotoxicity, although this also resulted in decreased anti-HCoV-NL63 activity. In addition, the introduction of methoxy functionalities into emodin did not seem to improve its anti-HCoV-NL63 activity; this was probably related to the lower solubility of these compounds in aqueous solutions.

In summary, we applied various synthetic routes to prepare a limited number of emodin analogues that allowed us to perform some initial studies aimed at improving the anti-HCoV-NL63 activity of emodin. Of particular importance was the establishment of SAR guidelines related to anti-HCoV-NL63 activity. The availability of new synthetic routes for the diversification of an emodin structure should allow the generation of new emodin analogues with significantly improved properties, such as increased solubility and, most importantly, stronger anti-HCoV-NL63 activity and reduced toxicity, so that a much wider therapeutic window can realistically be generated.

## 5. Patents

Patent pending (LU500249).

## Figures and Tables

**Table 1:**
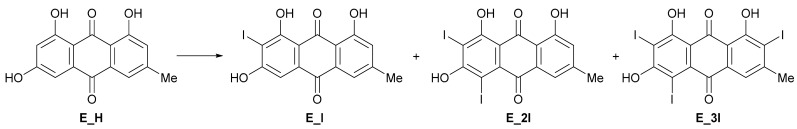
Iodination of emodin **E_H** with I_2_/NaHCO_3_, NIS or I_2_/H_2_O_2_.

Entry	Reagent (equiv.)	Solvent	Conditions	Relative Distribution ^a^			
				E_H	E_I	E_2I	E_3I
1	I_2_ (2), NaHCO_3_	THF/H_2_O	rt., 1 h	-	100 (73%)	-	-
2	I_2_ (4), NaHCO_3_	THF/H_2_O	rt., 1 h	-	100	-	-
3	I_2_ (10), NaHCO_3_	THF/H_2_O	rt., 24 h	-	-	-	100 (81%)
4	I_2_ (10), NaHCO_3_	THF/H_2_O	60 °C, 24 h	-	-	-	100
5	NIS (1)	THF	rt., 24 h	3	97	-	-
6	NIS (1.3)	THF	rt., 3 h	-	100 (84%)	-	-
7	NIS (1.3)	THF	rt., 24 h	-	93	7	-
8	NIS (2)	THF	rt., 24 h	-	86	14	-
9	NIS (4)	THF	rt., 24 h	-	-	56	44
10	NIS (4)	THF	60 °C, 24 h	-	-	-	100 (79%)
11	I_2_ (1)	2-MeTHF	rt., 24 h	100	-	-	-
12	I_2_ (0.5), H_2_O_2_ (8)	2-MeTHF	rt., 24 h	56	44	-	-
13	I_2_ (1), H_2_O_2_ (8)	2-MeTHF	rt., 24 h	21	79	-	-
14	I_2_ (1.5), H_2_O_2_ (2.5)	2-MeTHF	rt., 24 h	16	84	-	-
15	I_2_ (1.5), H_2_O_2_ (4)	2-MeTHF	rt., 24 h	13	87	-	-
16	I_2_ (2), H_2_O_2_ (2.5)	2-MeTHF	rt., 24 h	-	100 (93%)	-	-
17	I_2_ (8), H_2_O_2_ (8)	2-MeTHF	rt., 24 h	-	100	-	-

Reaction conditions: Emodin (0.1 mmol), reagent (I_2_ (0.2–1.0 mmol), NIS (*N*-iodosuccinimide) (0.1–0.4 mmol), H_2_O_2_ (30%, 0.25–0.8 mmol)), solvent (1 mL), ^a^ Conversion to product was determined by ^1^H NMR.

**Table 2:**
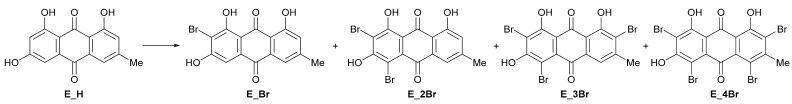
Bromination of emodin **E_H** with NBS and HBr/H_2_O_2_.

Entry	Reagent (Equiv.)	Solvent	Conditions	Relative Distribution ^a^				
				E_H	E_Br	E_2Br	E_3Br	E_4Br
1	NBS (1)	THF	0 °C, 10 min	34	40	26	-	-
2	NBS (1.5)	THF	0 °C, 15 min	17	43	40	-	-
3	NBS (2.2)	THF	0 °C, 30 min	-	-	100 (88%)	-	-
4	NBS (3)	THF	0 °C, 24 h	-	-	63	37	-
5	NBS (3)	THF	rt., 24 h	-	-	27	73	-
6	NBS (4)	THF	rt., 24 h	-	-	-	60	40
7	NBS (5)	THF	rt., 24 h	-	-	-	-	100 (83%)
8	HBr (1), H_2_O_2_ (2.5)	TFE	0 °C, 2 h	29	71	-	-	-
9	HBr (1.3), H_2_O_2_ (5)	TFE	0 °C, 2 h	67	31	2	-	-
10	HBr (2.5), H_2_O_2_ (5)	TFE	0 °C, 2 h	8	81	11	-	-
11	HBr (1), H_2_O_2_ (5)	TFE	rt., 24 h	9	79	12	-	-
12	HBr (2), H_2_O_2_ (5)	TFE	rt., 24 h	-	28	72	-	-
13	HBr (2.4), H_2_O_2_ (5)	TFE	rt., 24 h	-	14	86	-	-
14	HBr (4), H_2_O_2_ (5)	TFE	rt., 24 h	-	-	100 (91%)	-	-

Reaction conditions: Emodin (0.1 mmol), reagent (NBS (*N*-bromosuccinimide) (0.1–0.5 mmol), HBr (48%, 0.1–0.4 mmol), H_2_O_2_ (30%, 0.25–0.5 mmol)), solvent (1 mL). ^a^ Conversion to product was determined by ^1^H NMR.

**Table 3:**
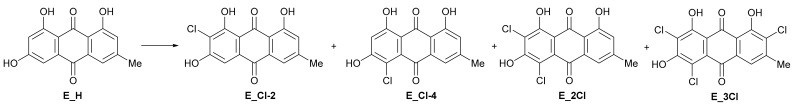
Chlorination of emodin **E_H** with NCS and HCl/H_2_O_2_.

Entry	Reagent (Equiv.)	Solvent	Conditions	Relative Distribution ^a^			
				E_H	E_Cl	E_2Cl	E_3Cl
1	NCS (1)	THF	rt., 24 h	100	-	-	-
2	NCS (2)	THF	rt., 24 h	49	42	9	-
3	NCS ^b^ (2.5)	THF	rt., 3 h	-	-	100 (81%)	-
4	NCS ^b^ (6)	THF	60 °C, 24 h	-	-	100	-
5	HCl (1), H_2_O_2_ (2)	TFE	rt., 24 h	56	36	8	-
6	HCl (2), H_2_O_2_ (5)	TFE	rt., 24 h	10	68	22	-
7	HCl (3), H_2_O_2_ (5)	TFE	rt., 24 h	-	44	39	17
8	HCl (4), H_2_O_2_ (5)	TFE	rt., 24 h	-	41	37	22
9	HCl (5), H_2_O_2_ (10)	TFE	rt., 24 h	-	17	50	33

Reaction conditions: Emodin (0.1 mmol), reagent (NCS (*N*-chlorosuccinimide) (0.1–0.6 mmol), HCl (37%, 0.1–0.5 mmol), H_2_O_2_ (30%, 0.2–1.0 mmol)), solvent (1 mL), ^a^ Conversion to product was determined by ^1^H NMR, ^b^ H_2_SO_4_.

**Table 4:** Nitration of emodin with HNO_3_ and H_2_SO_4_.

Entry	Reagent (Equiv.) ^a^	Conditions	Conv. ^b^	
			E_X	E_4NO_2_
1	HNO_3_ (1), H_2_SO_4_ (4), MeCN	0 °C, 1 h	2%	-
2	HNO_3_ (2.5), H_2_SO_4_ (10)	0 °C, 1 h	78%	22%
3	HNO_3_ (2.5), H_2_SO_4_ (20)	0 °C, 1 h	79%	21%
4	HNO_3_ (3), H_2_SO_4_ (10)	0 °C, 1 h	59%	41%
5	HNO_3_ (3), H_2_SO_4_ (20)	0 °C, 1 h	27%	73%
6	HNO_3_ (4.4), H_2_SO_4_ (20)	0 °C, 1 h	10%	90%
7	HNO_3_ (6.2), H_2_SO_4_ (5)	0 °C, 0.25 h	13%	87%
8	HNO_3_ (6.2), H_2_SO_4_ (10)	0 °C, 0.5 h	-	100 (91%)
9	HNO_3_ (63), H_2_SO_4_ (80)	0 °C, 1 h; rt, 4 h	-	100 % [24]

^a^ Emodin (0.1 mmol), reagent (HNO_3_ (0.1–6.3 mmol), H_2_SO_4_ (0.4–8.0 mmol)), MeCN/no solvent, ^b^ Conversion to product was determined by ^1^H NMR relative to **E_H**. **E_X** is a mixture of mono-, di- and trinitroemodin.

**Table 5:** IC_50_ (µM) values corresponding to the anti HCoV-NL63 effects (AV) and the effects on Vero cell viability (expressed as IC_50_ values, CV) of each of the tested compounds.

	IC_50_ (µM)	
	Exp.	CT
**E_H**	2.5	4.9
**E_4NO_2_**	6.1	6.1
**E_I**	1.3	3.6
**E_3I**	0.5	4.9
**E_4Br**	1.7	7.2
**E_2Br**	1.0	5.4
**E_2Cl**	1.1	7.5
**E_OMe**	>50	8.7
**E_SO_3_H**	22.0	>50
**E_NH_2_**	6.3	41.8
**E_Br_OMe**	>50	>50
Remdesivir	0.61	
Chloroquine	19.2	

## Data Availability

The data present in this study are available in Supplementary Materials.

## Supplementary Materials

The following are available online. Figure S1: Chlorination of emodin **E_H** with NCS and HCl/H_2_O_2_, Figure S2: Characterization of **E_Cl-2** with ^1^H NMR and 2D (HSQC, HMBC) NMR in DMSO, 500 MHz, Figure S3: Characterization of **E_Cl-4** with ^1^H and 2D (HSQC, HMBC) NMR in DMSO, 500 MHz, Figure S4: Following a desulfonation of trisubstituted emodin **E_3SO_3_H** to monosubstituted **E_SO_3_H** in MeCN/hexane at room temperature by ^1^H NMR (bottom spectra: crude reaction mixture, middle spectra: after 1 h, top spectra: after 3 h), Figure S5: IC_50_ curves for anti-HCoV-NL63 effects of standard compounds chloroquine and Remdesivir, Figure S6: ^1^H NMR spectrum of compound **E_I** in DMSO, 500 MHz, Figure S7: ^13^C NMR spectrum of compound **E_I** in DMSO, 500 MHz, Figure S8: 2D HSQC NMR spectrum of compound **E_I** in DMSO, 500 MHz, Figure S9: 2D HMBC NMR spectrum of compound **E_I** in DMSO, 500 MHz, Figure S10: ^1^H NMR spectrum of compound **E_3I** in DMSO, 500 MHz, Figure S11: ^13^C NMR spectrum of compound **E_3I** in DMSO, 500 MHz, Figure S12: 2D HSQC NMR spectrum of compound **E_3I** in DMSO, 500 MHz, Figure S13: 2D HMBC NMR spectrum of compound **E_3I** in DMSO, 500 MHz, Figure S14: ^1^H NMR spectrum of compound **E_2Br** in DMSO, 500 MHz, Figure S15: ^13^C NMR spectrum of compound **E_2Br** in DMSO, 500 MHz, Figure S16: 2D HSQC NMR spectrum of compound **E_2Br** in DMSO, 500 MHz, Figure S17: 2D HMBC NMR spectrum of compound **E_2Br** in DMSO, 500 MHz, Figure S18: ^1^H NMR spectrum of compound **E_4Br** in DMSO, 500 MHz, Figure S19: ^13^C NMR spectrum of compound **E_4Br** in DMSO, 500 MHz, Figure S20: ^1^H NMR spectrum of compound **E_2Cl** in DMSO, 500 MHz, Figure S21: ^13^C NMR spectrum of compound **E_2Cl** in DMSO, 500 MHz, Figure S22: 2D HSQC NMR spectrum of compound **E_2Cl** in DMSO, 500 MHz, Figure S23: 2D HMBC NMR spectrum of compound **E_2Cl** in DMSO, 500 MHz, Figure S24: ^1^H NMR spectrum of compound **E_4NO_2_** in DMSO, 500 MHz, Figure S25: ^13^C NMR spectrum of compound **E_4NO_2_** in DMSO, 500 MHz, Figure S26: ^1^H NMR spectrum of compound **E_NH_2_** in DMSO, 500 MHz, Figure S27: ^13^C NMR spectrum of compound **E_NH_2_** in DMSO, 500 MHz, Figure S28: ^1^H NMR spectrum of compound **E_SO_3_H** in DMSO, 500 MHz, Figure S29: ^13^C NMR spectrum of compound **E_SO_3_H** in DMSO, 500 MHz, Figure S30: 2D HSQC NMR spectrum of compound **E_SO_3_H** in DMSO, 500 MHz, Figure S31: 2D HMBC NMR spectrum of compound **E_SO_3_H** in DMSO, 500 MHz, Figure S32: 2D NOESY NMR spectrum of compound **E_SO_3_H** in DMSO, 500 MHz, Figure S33: ^1^H NMR spectrum of compound **E_OCH_3_** in CDCl_3_, 500 MHz, Figure S34: ^13^C NMR spectrum of compound **E_OCH_3_** in CDCl_3_, 500 MHz, Figure S35: ^1^H NMR spectrum of compound **E_Br_OCH_3_** in CDCl_3_, 500 MHz, Figure S36: ^13^C NMR spectrum of compound **E_Br_OCH_3_** in CDCl_3_, 500 MHz, Table S1: IC_50_ (µM) values indicating the effect of emodin and emodin analogues on Vero cell viability, Table S2: IC_50_ (µM) values for anti HCoV-NL63 effects of emodin and emodin analogs, Table S3: IC_50_ (µM) values for anti HCoV-NL63 effects of chloroquine and Remdesivir, Table S4: Percentage of live cells following exposure to various concentrations of emodin and emodin analogues, Table S5: Percentage of live cells following viral infection and exposure to various concentrations of emodin and emodin analogues, Table S6: Percentage of live cells following viral infection and exposure to various concentration of chloroquine, Table S7: Percentage of live cells following viral infection and exposure to various concentration of Remdesivir.

## Sample Availability

Samples of the compounds are available from the authors.
