# Supplementary material for: Antiviral Activities of Halogenated Emodin Derivatives against Human Coronavirus NL63
Source: Molecules. 2021 Nov 11;26(22):6825. doi: 10.3390/molecules26226825 (PMC8618202; doi:10.3390/molecules26226825)
Supplement: Supplementary file 1 [file molecules-26-06825-s001.zip › molecules-1455949-supplementary.pdf]

SUPPORTING INFORMATION

Antiviral Activities of Halogenated Emodin Derivatives against Human Coronavirus NL63

Monika Horvat [1], Martina Avbelj [2], María Beatriz Durán-Alonso [3], Mihailo Banjanac [4], Hrvoje Petković [2,*] and Jernej Iskra [1,*]

[1] *Faculty of Chemistry and Chemical Technology, University of Ljubljana, Večna pot 113, 1000 Ljubljana, Slovenia*

[2] *Biotechnical Faculty, University of Ljubljana, Jamnikarjeva 101, 1000 Ljubljana, Slovenia*

[3] *Unit of Excellence, Institute of Biology and Molecular Genetics (IBGM), University of Valladolid-CSIC, 47003 Valladolid, Spain*

[4] *Fidelta d.o.o., Prilaz baruna Filipovića 29, 10000 Zagreb, Croatia*

[*] Correspondence: hrvoje.petkovic@bf.uni-lj.si (H.P.); jernej.iskra@fkkt.uni-lj.si (J.I.)

TABLE OF CONTENTS

1. GENERAL INFORMATION 1

2. EXPERIMENTAL SECTION 1

Synthesis of emodin derivatives 1

Chlorination 1

Sulfonation 3

Synthesis and characterization of products 4

1,3,8-trihydroxy-2-iodo-6-methylanthracene-9,10-dione (E_I).[1] 4

2,4,5-trihydroxy-1,3,6-triiodo-7-methylanthracene-9,10-dione (E_3I).[1] 4

1,3-dibromo-2,4,5-trihydroxy-7-methylanthracene-9,10-dione (E_2Br).[1] 5

Antiviral activity of emodins 5

Antiviral activity raw data 7

Toxicity to Vero cells 7

Antiviral activity 7

Antiviral activity of chloroquine 8

Antiviral activity of Remdesivir 8

3. COPIES OF NMR SPECTRA 9

1,3,8-trihydroxy-2-iodo-6-methylanthracene-9,10-dione (E_I). 9

2,4,5-trihydroxy-1,3,6-triiodo-7-methylanthracene-9,10-dione (E_3I). 11

1,3-dibromo-2,4,5-trihydroxy-7-methylanthracene-9,10-dione (E_2Br). 13

1,3,6,8-tetrabromo-2,4,5-trihydroxy-7-methylanthracene-9,10-dione (E_4Br). 15

1,3-dichloro-2,4,5-trihydroxy-7-methylanthracene-9,10-dione (E_2Cl). 16

1,3,8-trihydroxy-6-methyl-2,4,5,7-tetranitroanthracene-9,10-dione (E_4NO_2). 18

1-((3-aminopropyl)amino)-2,4,5-trihydroxy-7-methylanthracene-9,10-dione (E_NH_2). 19

1,3,8-trihydroxy-6-methyl-9,10-dioxo-9,10-dihydroanthracene-2-sulfonic acid (E_SO_3H). 20

1,3,8-trimethoxy-6-methylanthracene-9,10-dione (E_OCH_3). 23

1,3,6,8-tetrabromo-2,4,5-trimethoxy-7-methylanthracene-9,10-dione (E_Br_OCH_3). 24

4. LITERATURE 25

1. GENERAL INFORMATION

Emodin purchased from Fluorochem was used as received. All other reagents and solvents were of reagent-grade quality and obtained from commercial suppliers (Honeywell, Sigma-Aldrich). TLC was performed on Merck-60-F254 plates using mixtures of EtOAc:EtOH (10:1), CH2Cl2:EtOH (100:1) or EtOAc:MeOH (20:1). Crude products were purified by column chromatography on silica gel (63-200 µm, 70-230 mesh ASTM; Fluka). The products were characterized by 1H, 13C NMR spectra, HRMS and IR analysis. 1H and 13C NMR spectra were recorded on Bruker Avance III 500 instruments, IR spectra were recorded on Spectrum BX FTIR Perkin-Elmer. HR-MS were recorded on LC MS system Agilent 6224 Accurate Mass TOF LC/MS.

2. EXPERIMENTAL SECTION

Synthesis of emodin derivatives

Chlorination

We found out that when the NCS reagent was used, the Cl atom was bonded to side 2 (in HMBC 2D NMR spectra correlation between carbonyl C-10 atom and H-4 and H-5 is indicative as well as methyl C-atom with H-5 and H-7) and when HCl/H_2O_2 was used, the Cl atom was bonded to side 4 (in HMBC 2D NMR spectra carbonyl C-10 atom is correlated only with H-5, while methyl C-atom is correlated with H-5 and H-7).

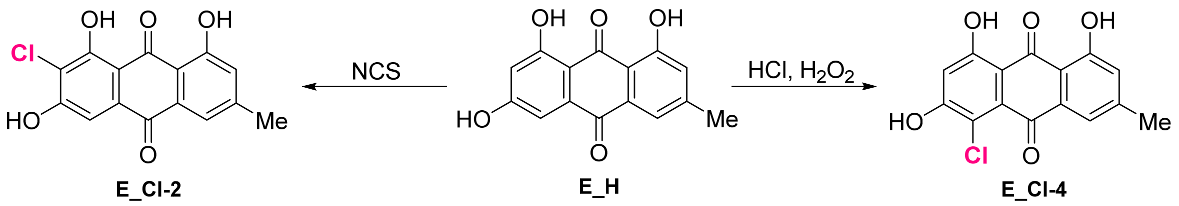

Figure S1. *Chlorination of emodin E_H with NCS and HCl/H_2O_2.*

E_Cl-2: ^{1}H NMR (500 MHz, DMSO-d_6, 25 °C): δ = 7.49 (s, 1H, ArH), 7.35 (s, 1H, ArH), 7.18 (s, 1H, ArH), 2.41 (s, 3H, CH_3) ppm.

E_Cl-4: ^{1}H NMR (500 MHz, DMSO-d_6, 25 °C): δ = 7.44 (s, 1H, ArH), 7.13 (s, 1H, ArH), 6.78 (s, 1H, ArH), 2.41 (s, 3H, CH_3) ppm.

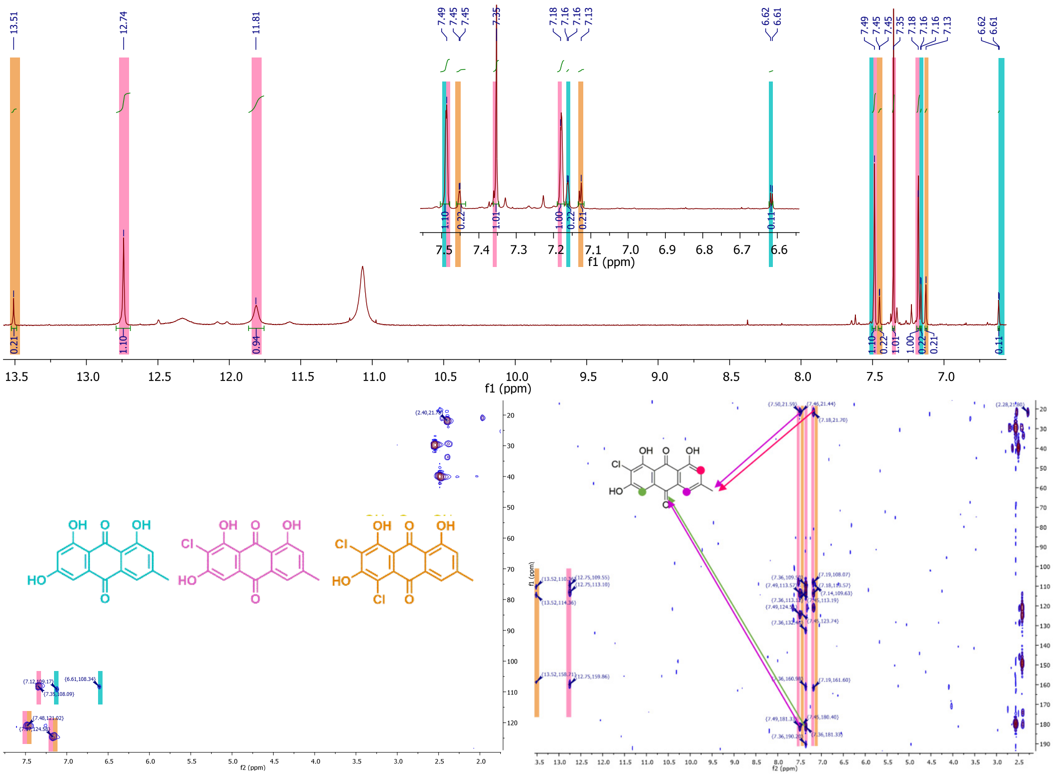

Figure S2. *Characterization of E_Cl-2 with ^{1}H NMR and 2D (HSQC, HMBC) NMR in DMSO, 500 MHz.*

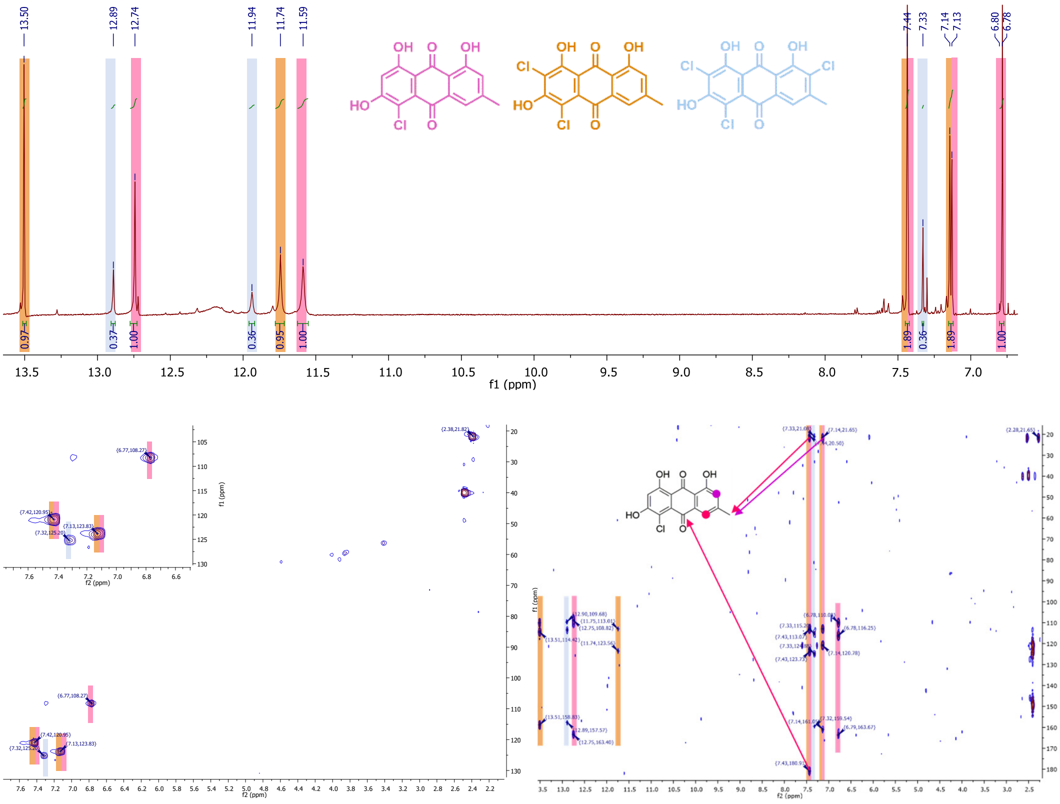

Figure S3. *Characterization of E_Cl-4 with ^{1}H and 2D (HSQC, HMBC) NMR in DMSO, 500 MHz.*

Sulfonation

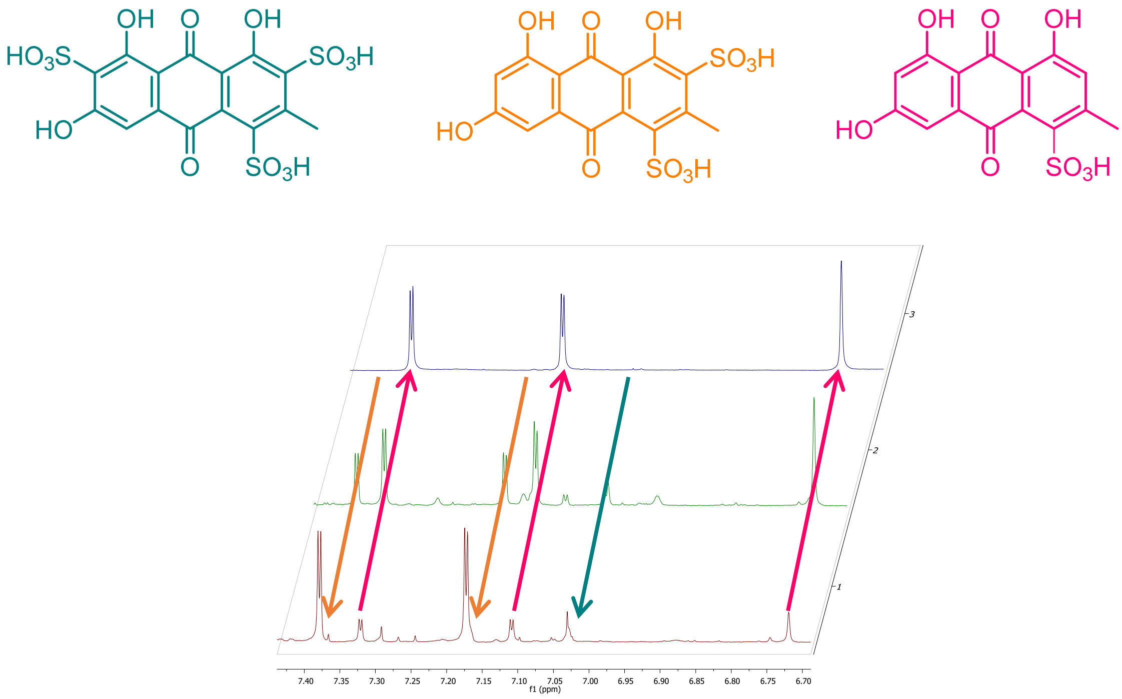

Figure S4. *Following a desulfonation of trisubstituted emodin E_3SO$_3$H to monosubstituted E_SO$_3$H in MeCN/hexane at room temperature by ^{1}H NMR (bottom spectra: crude reaction mixture, middle spectra: after 1 h, top spectra: after 3 h).*

Synthesis and characterization of products

1,3,8-trihydroxy-2-iodo-6-methylanthracene-9,10-dione (E_I).[1]

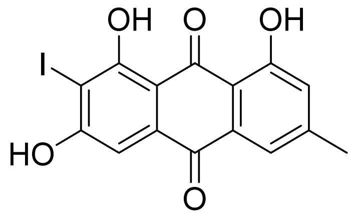

<u>Synthesis procedure 1:</u> Iodine (254 mg, 1.0 mmol) was added at a temperature of 0 °C to a stirred solution of emodin (135 mg, 0.5 mmol) in THF (5 mL) and water (5 mL). Then $NaHCO_3$ (310 mg, 3.7 mmol) was added in a stepwise manner. The reaction mixture was stirred at room temperature for 1 h. The reaction was monitored by TLC (CH_2Cl_2:EtOH=100:1). After the reaction was complete, the mixture was extracted with dichloromethane (3×20 mL). The organic layer was washed with water (3×20 mL), dried over anhydrous Na_2SO_4 and evaporated under vacuum. The crude reaction product was washed with hexane (5 mL) and acetonitrile (5 mL) to remove soluble impurities. The product was dried in vacuum to provide the product (144 mg, 73%) as an orange solid.

<u>Synthesis procedure 2:</u> NIS (146 mg, 0.65 mmol) was added to a stirred solution of emodin (135 mg, 0.5 mmol) in THF (5 mL). The reaction mixture was stirred at room temperature for 3 h. The reaction was monitored by TLC (CH_2Cl_2:EtOH=100:1). After the reaction was complete, the mixture was extracted with dichloromethane (3×20 mL). The organic layer was washed with water (3×20 mL), dried over anhydrous Na_2SO_4 and evaporated under vacuum. The crude reaction product was washed with hexane (5 mL) and acetonitrile (5 mL) to remove soluble impurities. The product was dried in vacuum to provide the product (166 mg, 84%) as an orange solid.

1,3,8-trihydroxy-2,4,7-triiodo-6-methylanthracene-9,10-dione (E_3I).[1]

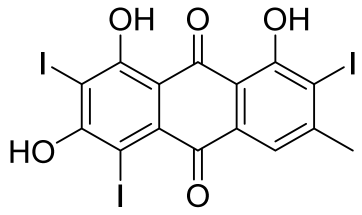

NIS (450 mg, 2.0 mmol) was added to a stirred solution of emodin (135 mg, 0.5 mmol) in THF (5 mL). The reaction mixture was stirred at 60 °C for 24 h. The reaction was monitored by TLC (CH_2Cl_2:EtOH=100:1). After the reaction was complete, the mixture was extracted with dichloromethane (3×20 mL). The organic layer was washed with water (3×20 mL), dried over anhydrous Na_2SO_4 and evaporated under vacuum. The crude reaction product was washed with hexane (5 mL) and acetonitrile (5 mL) to remove soluble impurities. The product was dried in vacuum to provide the product (256 mg, 79%) as an orange solid.

2,4-dibromo-1,3,8-trihydroxy-6-methylanthracene-9,10-dione (E_2Br).[1]

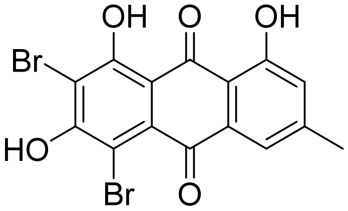

N-Bromosuccinimide (196 mg, 1.1 mmol) was added to a solution of emodin (135 mg, 0.5 mmol) in THF (5 mL) and stirred for 30 min at 0 °C. The reaction was monitored by TLC (CH_2Cl_2:EtOH = 100:1). After the reaction was complete, the mixture was extracted with CH_2Cl_2 (3×30 mL). The organic layer was dried over anhydrous Na_2SO_4 and evaporated under vacuum. The crude reaction product was washed with hexane (5 mL) and acetonitrile (3×5 mL) to remove soluble impurities. The product was dried in vacuum to provide the product (188 mg, 88%) as a yellow–orange solid.

Antiviral activity of emodins

Table S1. *IC_{50} (µM) values indicating the effect of emodin and emodin analogues on Vero cell viability.*

	E_H	E_NO_2	E_I	E_3I	E_4Br	E_2Br	E_Cl	E_OMe	E_SO_3H	E_NH_2	E_Br_OMe
**IC_{50} (µM)**	**4.9**	**6.1**	**3.6**	**4.9**	**7.2**	**5.4**	**7.5**	**8.7**	**>50**	**41.8**	**>50**
**Bottom**	1	1	4	5	4	7	6	= 0.000	= 0.000	= 0.000	n.a.
**Top**	118	108	92	93	94	99	104	121	111	105	n.a.
**HillSlope**	-3.8	-3.1	~ -23.63	-5.5	-5.8	-3.3	-4.6	-0.8	-1.8	-2.1	n.a.
**R squared**	1.00	1.00	1.00	1.00	0.98	0.99	0.97	0.92	0.85	0.94	n.a.

Table S2. *IC_{50} (µM) values for anti HCoV-NL63 effects of emodin and emodin analogs.*

	E_H	E_NO_2	E_I	E_3I	E_4Br	E_2Br	E_Cl	E_OMe	E_SO_3H	E_NH_2	E_Br_OMe
**IC_{50} (µM)**	**2.5**	**6.1**	**1.3**	**0.5**	**1.7**	**1.0**	**1.1**	**>50**	**22.0**	**6.3**	**>50**
**Bottom**	= 0.000	= 0.000	= 0.000	= 0.000	= 0.000	= 0.000	= 0.000	n.a.	= 0.000	= 0.000	n.a.
**Top**	= 100.0	= 100.0	= 100.0	= 100.0	= 100.0	= 100.0	= 100.0	n.a.	= 100.0	= 100.0	n.a.
**HillSlope**	1.206	2.138	1.308	1.597	1.628	2.434	1.339	n.a.	1.639	1.32	n.a.
**R squared**	0.89	0.96	0.99	0.97	0.97	0.99	0.97	n.a.	0.95	0.97	n.a.

Table S3. *IC_{50} (µM) values for anti HCoV-NL63 effects of chloroquine and Remdesivir.*

	Chloroquine	Remdesivir
**IC_{50} (µM)**	**19.2**	**0.61**
**Bottom**	= 0.000	= 0.000
**Top**	= 100.0	= 100.0
**HillSlope**	1.58	2.16
**R squared**	0.90	0.99

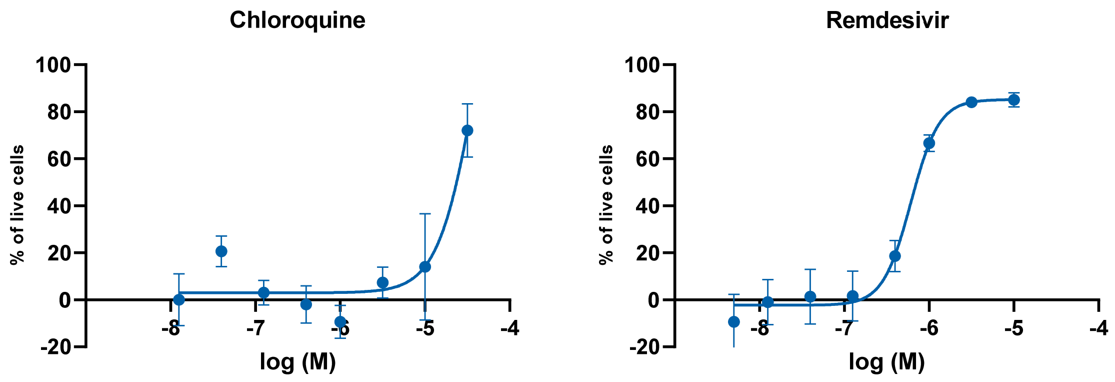

Figure S5. *IC_{50} curves for anti-HCoV-NL63 effects of standard compounds chloroquine and Remdesivir.*

Antiviral activity raw data

Toxicity to Vero cells

Table S4. Percentage of live cells following exposure to various concentrations of emodin and emodin analogues

Conc. (uM)	E_H	E_NO$_2$	E_I	E_3I	E_4Br	E_2Br	E_Cl	E_OMe	E_SO$_3$H	E_NH$_2$	E_Br_OMe
**50**	3	3	9	9	3	11	10	38	67	46	108
**25**	0	2	1	3	7	8	6	34	82	71	102
**12.5**	2	11	1	2	5	5	11	39	115	106	105
**6.25**	34	54	3	23	68	44	77	68	115	105	104
**3.13**	100	96	88	86	82	83	85	85	113	106	102
**1.56**	113	103	92	89	94	97	113	109	111	108	102
**0.78**	118	106	90	92	92	93	106	104	108	102	99
**0.39**	120	113	93	98	108	106	109	105	103	99	106
**IC$_{50}$ (μM)**	4.9	6.1	3.6	4.9	7.2	5.4	7.5	8.7	>50	41.8	>50
**Bottom**	1	1	4	5	4	7	6	= 0.000	= 0.000	= 0.000	n.a.
**Top**	118	108	92	93	94	99	104	121	111	105	n.a.
**HillSlope**	-3.8	-3.1	~ -23.63	-5.5	-5.8	-3.3	-4.6	-0.8	-1.8	-2.1	n.a.
**R squared**	1.00	1.00	1.00	1.00	0.98	0.99	0.97	0.92	0.85	0.94	n.a.

Antiviral activity

Table S5. Percentage of live cells following viral infection and exposure to various concentrations of emodin and emodin analogues

Conc. (uM)	E_H	E_NO2	E_I	E_3I	E_4Br	E_2Br	E_Cl	E_OMe	E_SO3H	E_NH2	E_Br_OMe
**50**	-125	120	15	-17	90	5	-39	1	85	13	-21
**25**	-130	46	-63	-30	-65	-80	-168	-7	51	77	-17
**12.5**	-133	84	-143	-122	-45	-72	-118	-23	23	77	-21
**6.25**	45	52	-13	-118	59	-72	83	-1	26	51	-6
**3.13**	61	15	74	91	68	54	82	-26	-3	28	-27
**1.56**	27	13	58	86	52	73	64	-10	-2	13	-17
**0.78**	27	3	37	74	19	37	39	-6	2	6	-30
**0.39**	9	10	14	41	5	6	16	-10	-2	-5	-33
**IC$_{50}$ (μM)**	2.5	6.1	1.3	0.5	1.7	1.0	1.1	>50	22.0	6.3	>50
**Bottom**	= 0.000	= 0.000	= 0.000	= 0.000	= 0.000	= 0.000	= 0.000	n.a.	= 0.000	= 0.000	n.a.

**Top**	= 100.0	= 100.0	= 100.0	= 100.0	= 100.0	= 100.0	= 100.0	n.a.	= 100.0	= 100.0	n.a.
**HillSlope**	1.206	2.138	1.308	1.597	1.628	2.434	1.339	n.a.	1.639	1.32	n.a.
**R squared**	0.89	0.96	0.99	0.97	0.97	0.99	0.97	n.a.	0.95	0.97	n.a.

Antiviral activity of chloroquine

Table S6. Percentage of live cells following viral infection and exposure to various concentration of chloroquine

Conc. (uM)	Chloroquine
30	77
10	14
3.333	7
1.111	-9
0.370	-2
0.123	3
0.041	20
0.014	0
IC50	19.2
Bottom	= 0.000
Top	= 100.0
HillSlope	1.58
R squared	0.90

Antiviral activity of Remdesivir

Table S7. Percentage of live cells following viral infection and exposure to various concentration of Remdesivir

Conc. (uM)	Remdesivir
10	85
3.333	84
1.111	67
0.370	19
0.123	2
0.041	1
0.014	-1
0.005	-9
IC50	0.61
Bottom	-3
Top	86
HillSlope	2.16
R squared	0.99

3. COPIES OF NMR SPECTRA

1,3,8-trihydroxy-2-iodo-6-methylanthracene-9,10-dione (E_I).

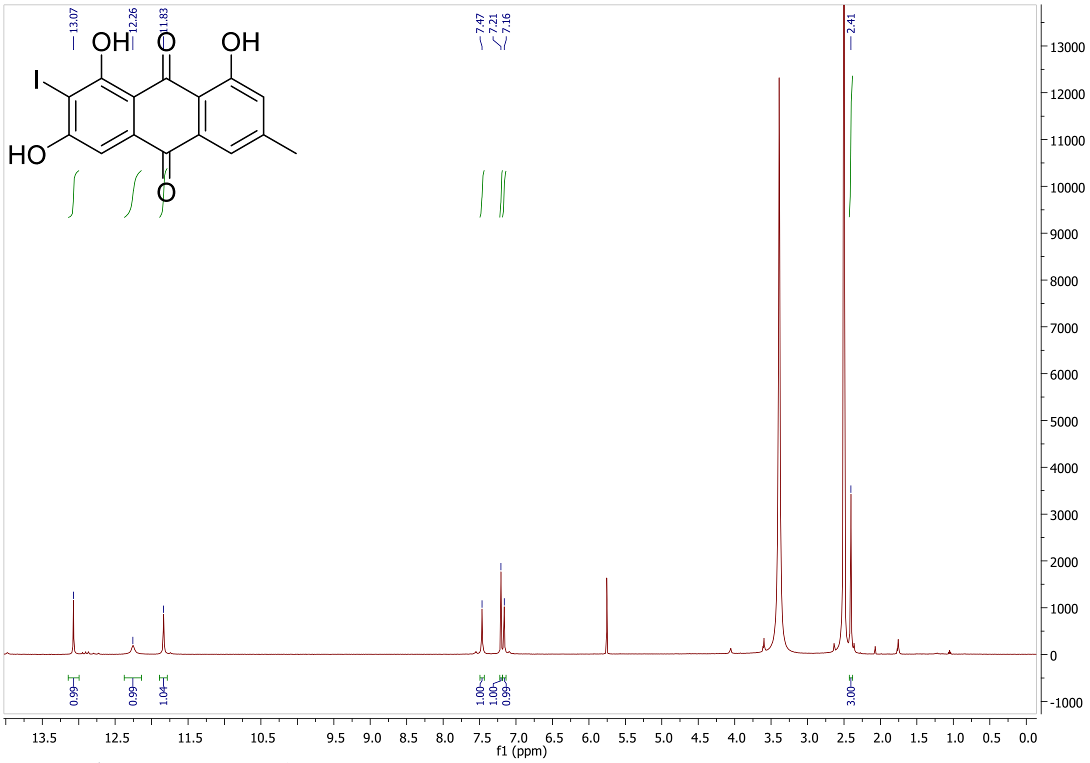

Figure S6. *^{1}H NMR spectrum of compound E_I in DMSO, 500 MHz.*

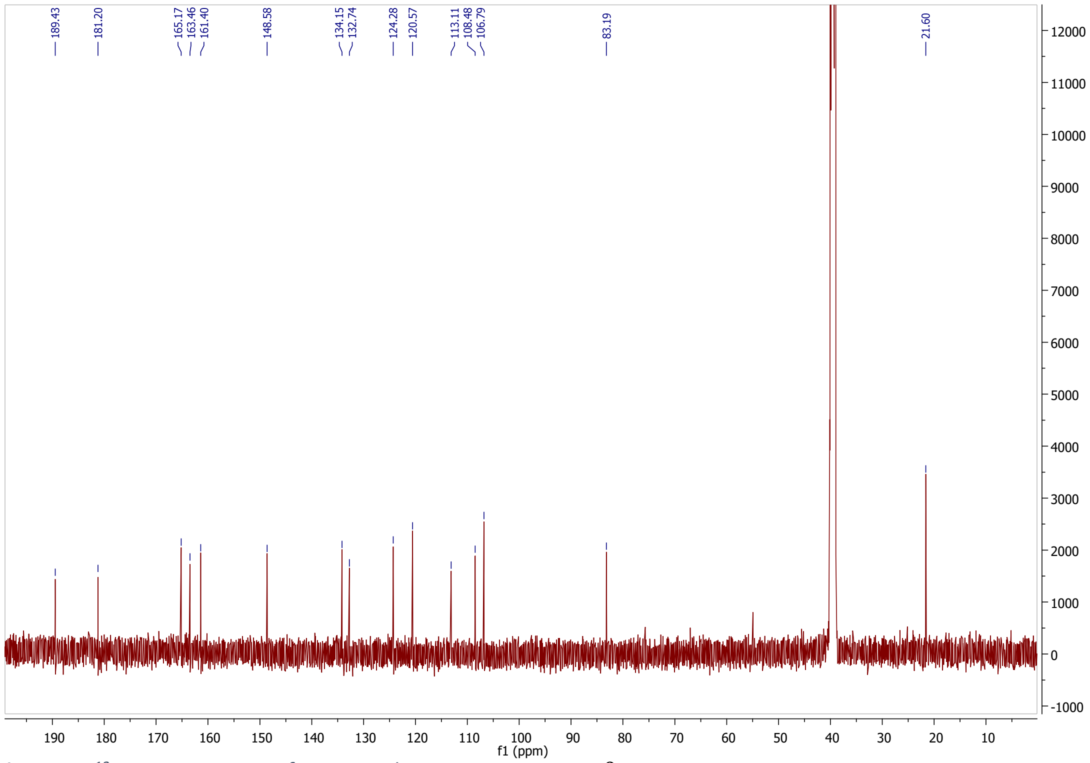

Figure S7. *^{13}C NMR spectrum of compound E_I in DMSO, 500 MHz.*

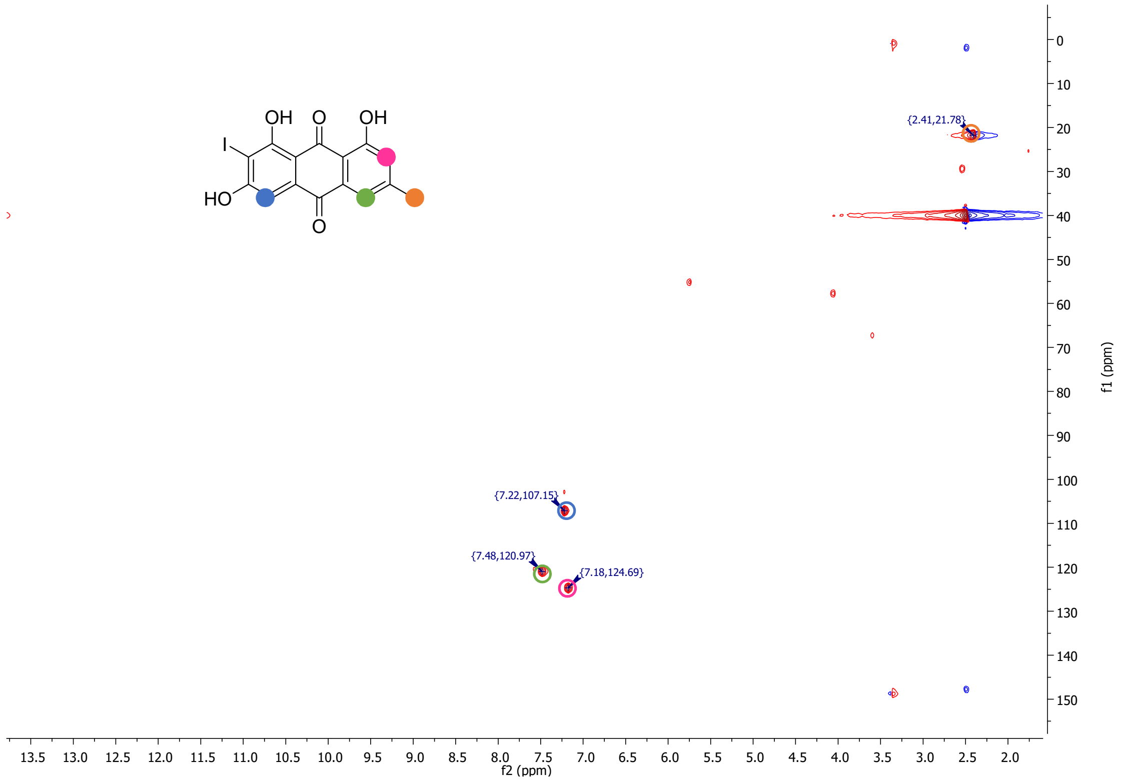

Figure S8. *2D HSQC NMR spectrum of compound E_I in DMSO, 500 MHz.*

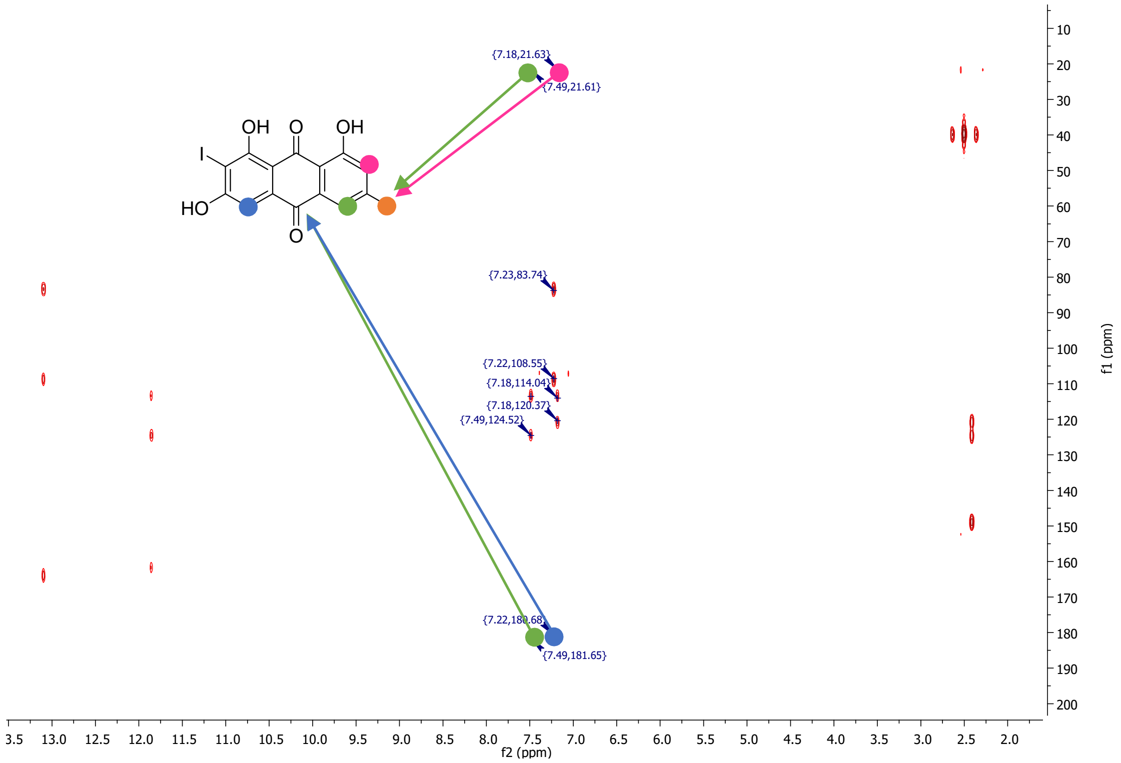

Figure S9. *2D HMBC NMR spectrum of compound E_I in DMSO, 500 MHz.*

1,3,8-trihydroxy-2,4,7-triiodo-6-methylanthracene-9,10-dione (E_3I).

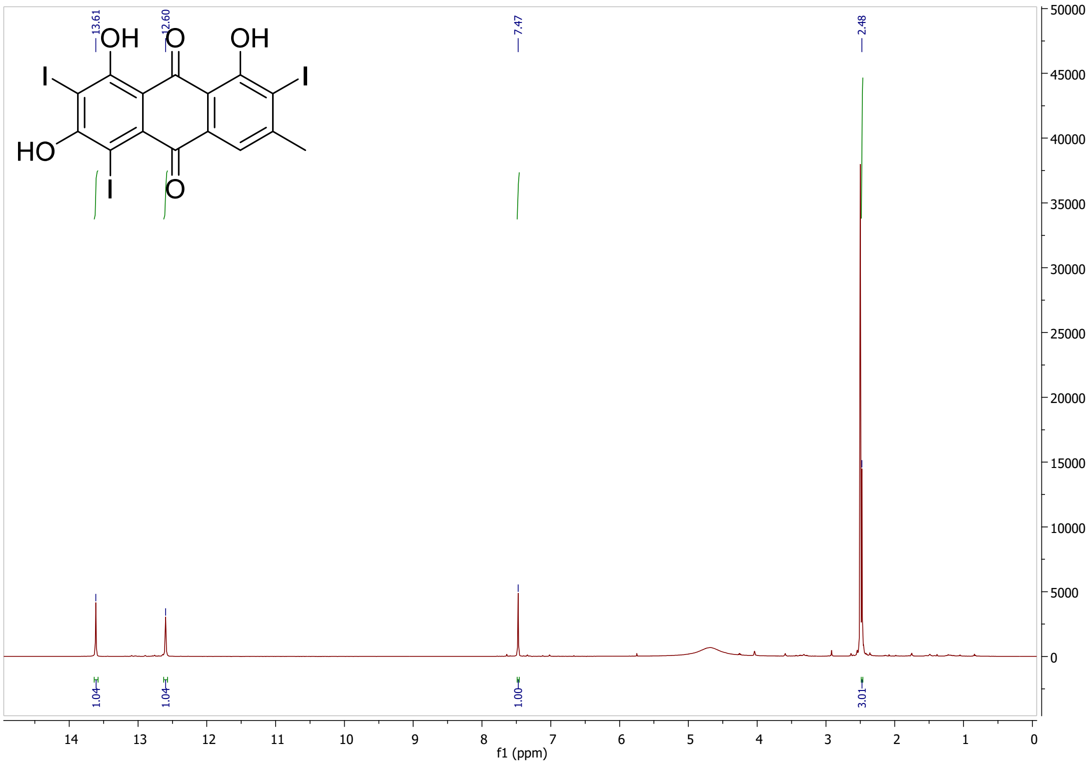

Figure S10. *^{1}H NMR spectrum of compound E_3I in DMSO, 500 MHz.*

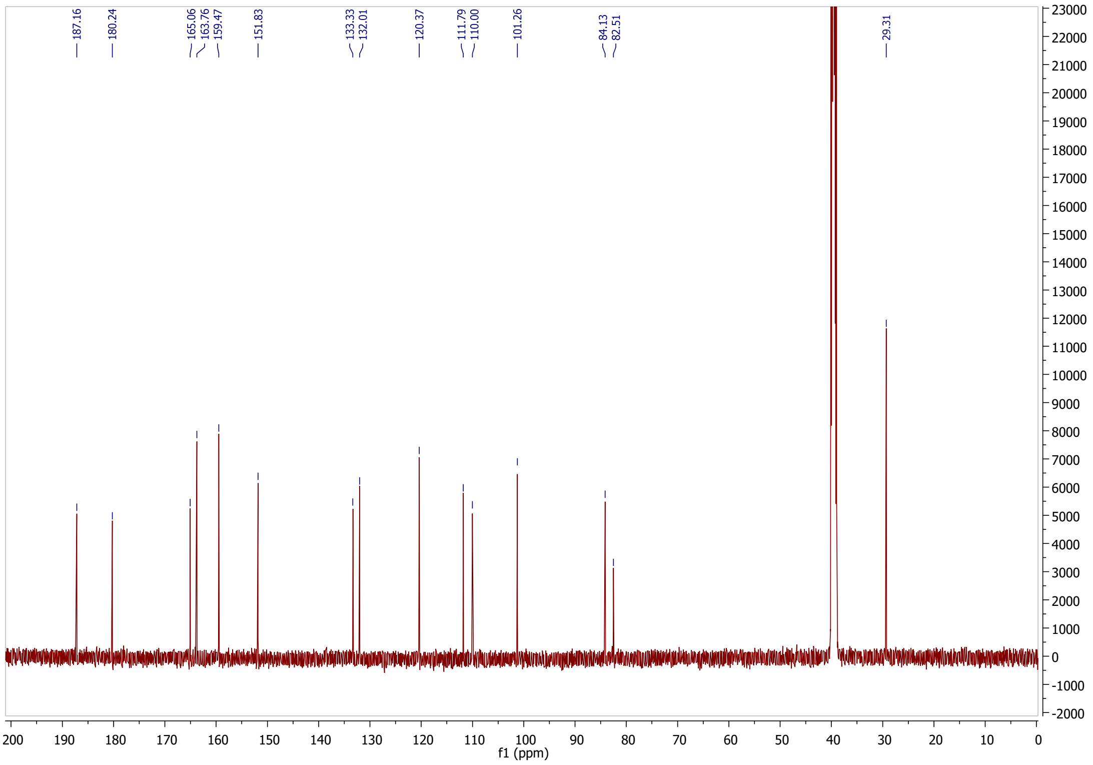

Figure S11. *^{13}C NMR spectrum of compound E_3I in DMSO, 500 MHz.*

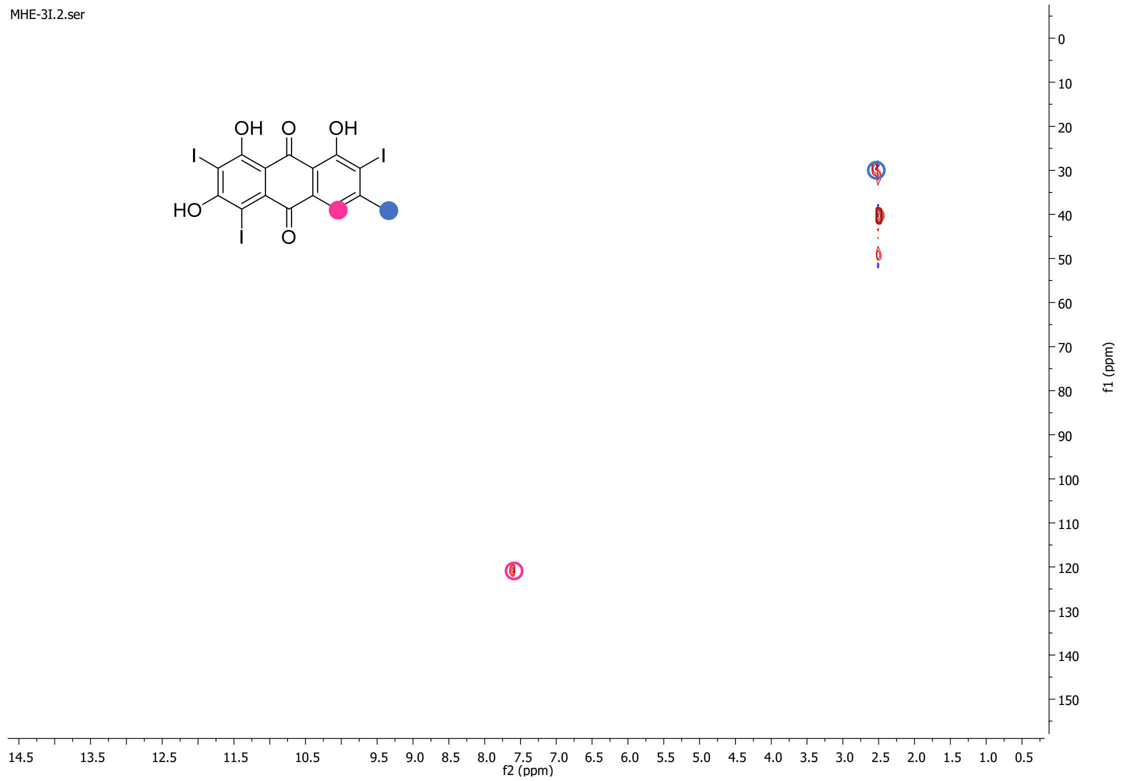

Figure S12. *2D HSQC NMR spectrum of compound E_3I in DMSO, 500 MHz.*

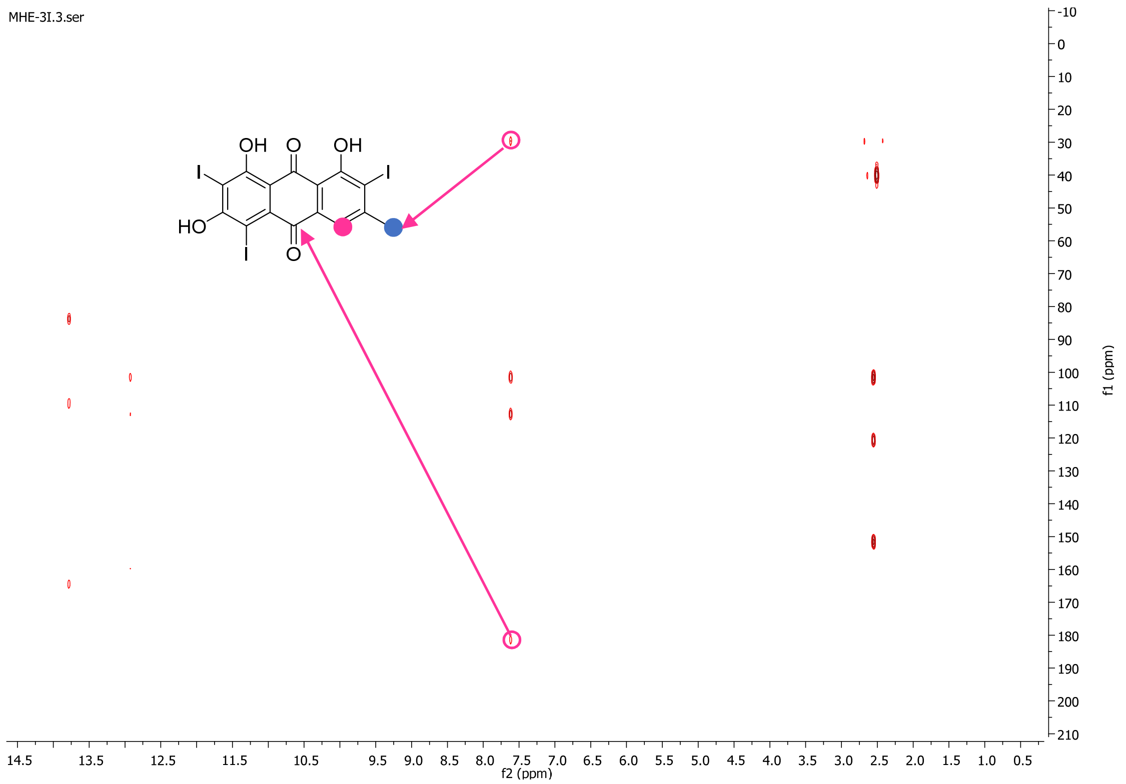

Figure S13. *2D HMBC NMR spectrum of compound E_3I in DMSO, 500 MHz.*

2,4-dibromo-1,3,8-trihydroxy-6-methylanthracene-9,10-dione (E_2Br).

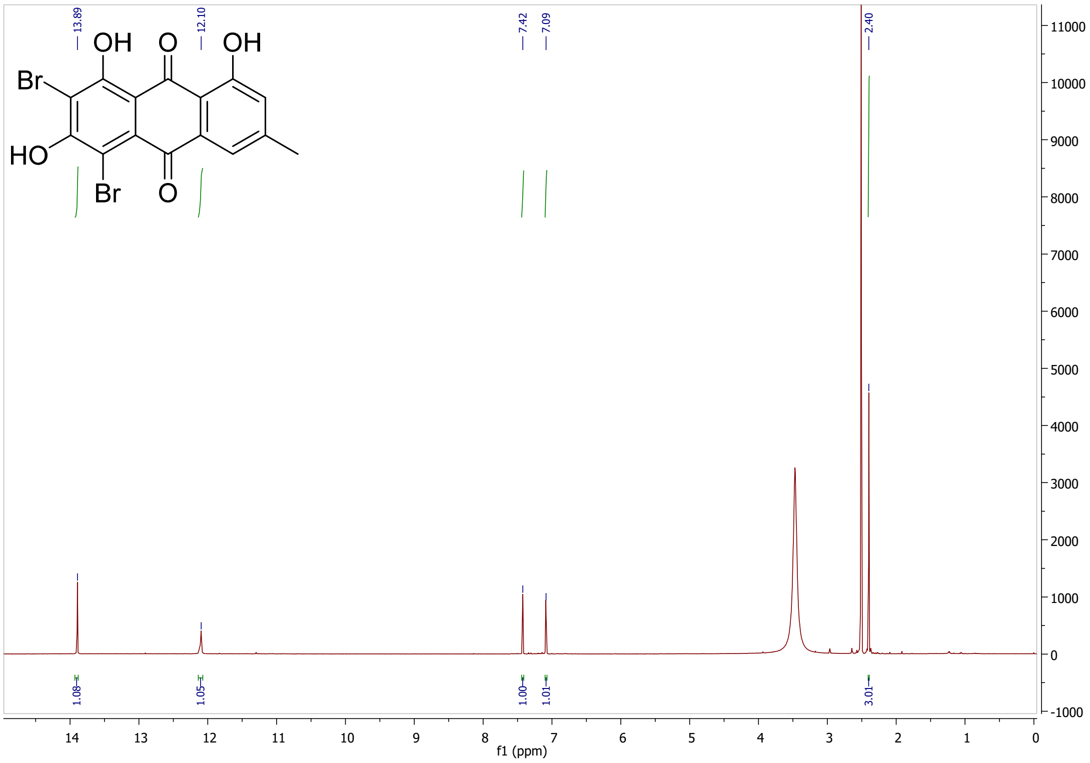

Figure S14. *^{1}H NMR spectrum of compound E_2Br in DMSO, 500 MHz.*

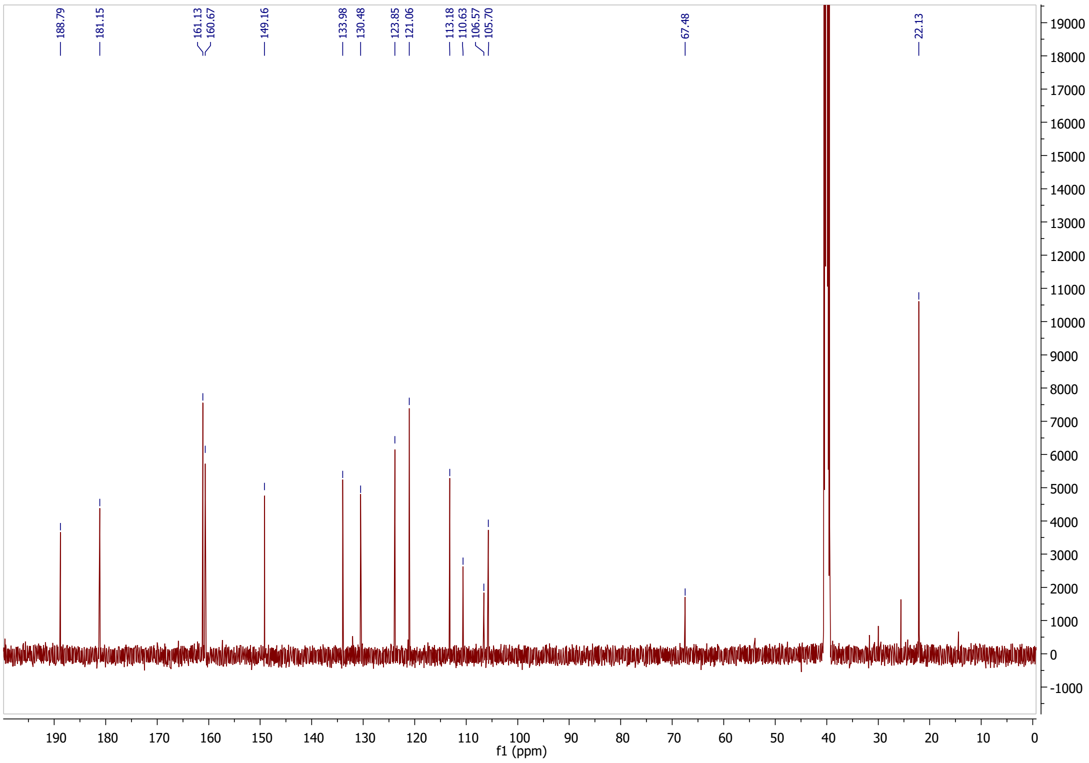

Figure S15. *^{13}C NMR spectrum of compound E_2Br in DMSO, 500 MHz.*

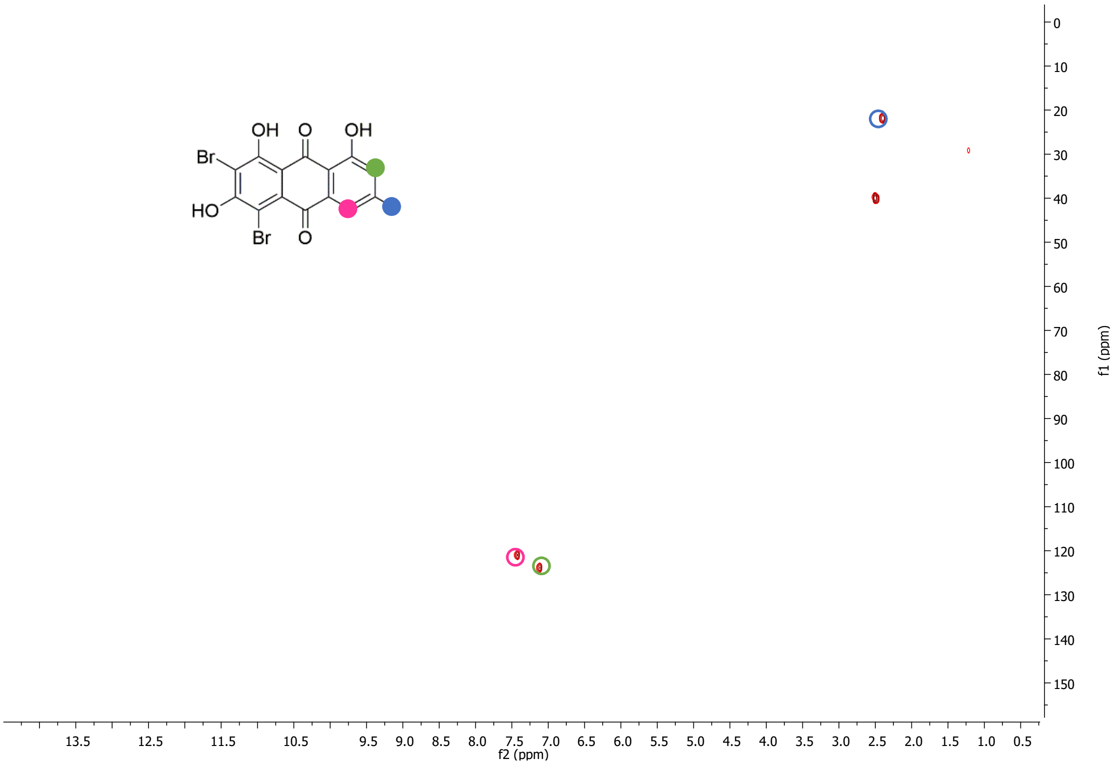

Figure S16. *2D HSQC NMR spectrum of compound E_2Br in DMSO, 500 MHz.*

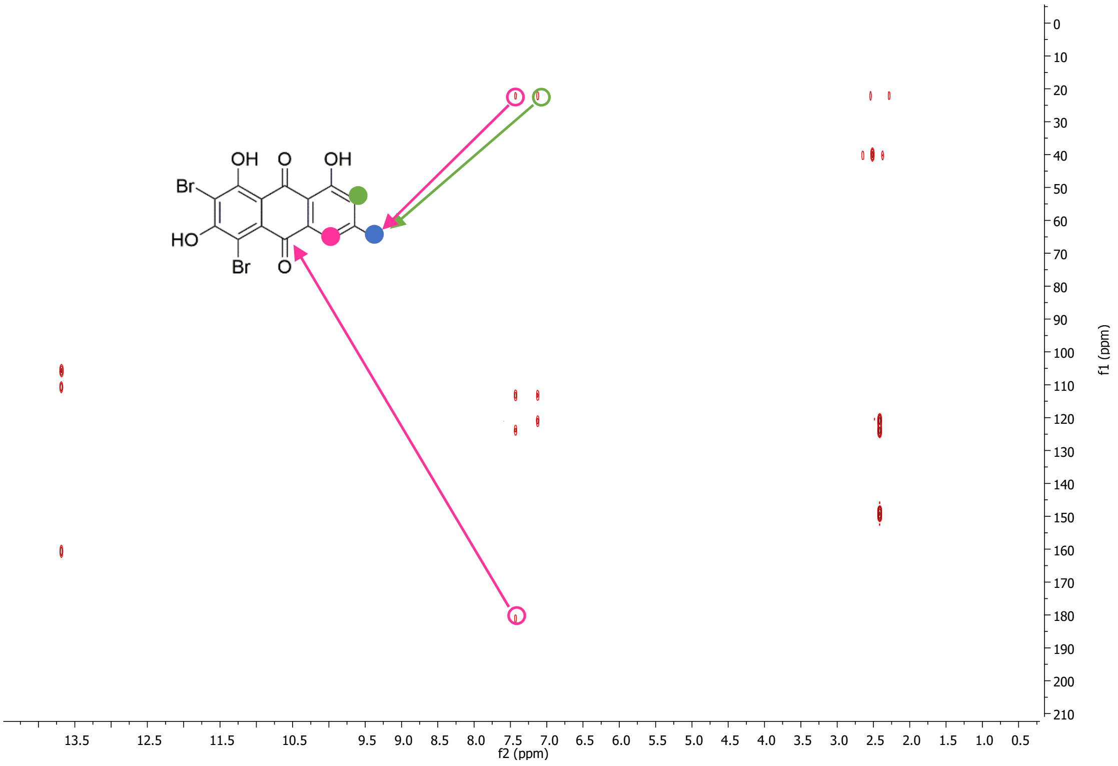

Figure S17. *2D HMBC NMR spectrum of compound E_2Br in DMSO, 500 MHz.*

2,4,5,7-tetrabromo-1,3,8-trihydroxy-6-methylanthracene-9,10-dione (E_4Br).

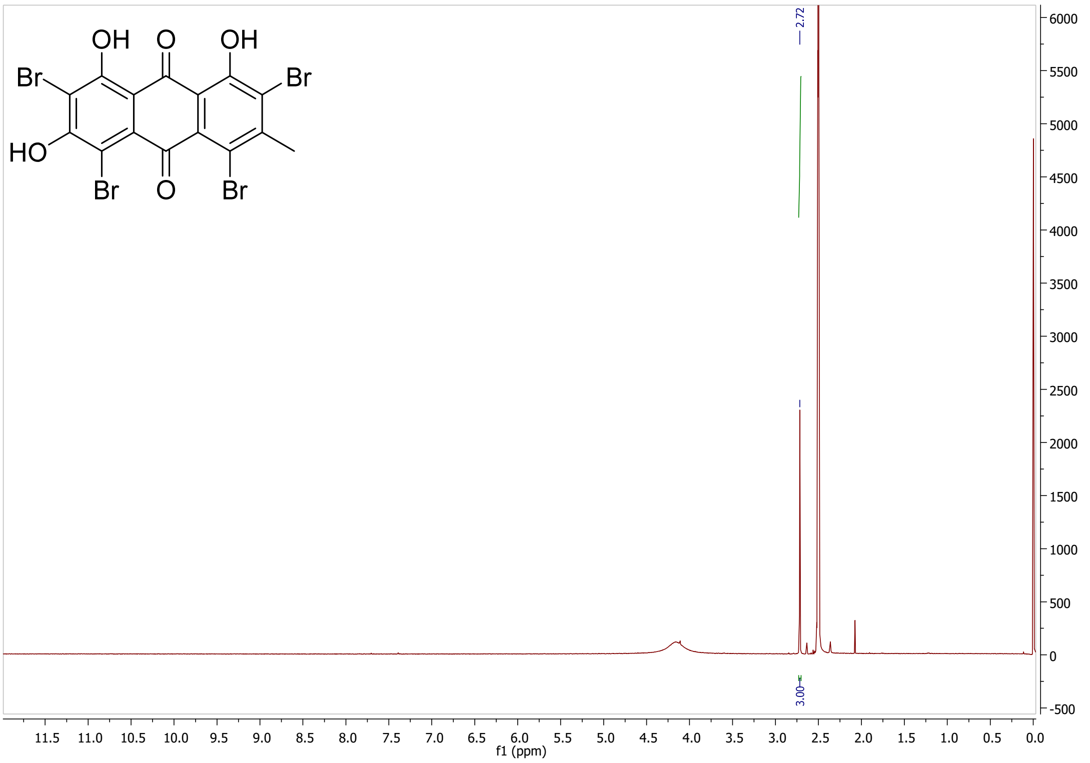

Figure S18. *^{1}H NMR spectrum of compound E_4Br in DMSO, 500 MHz.*

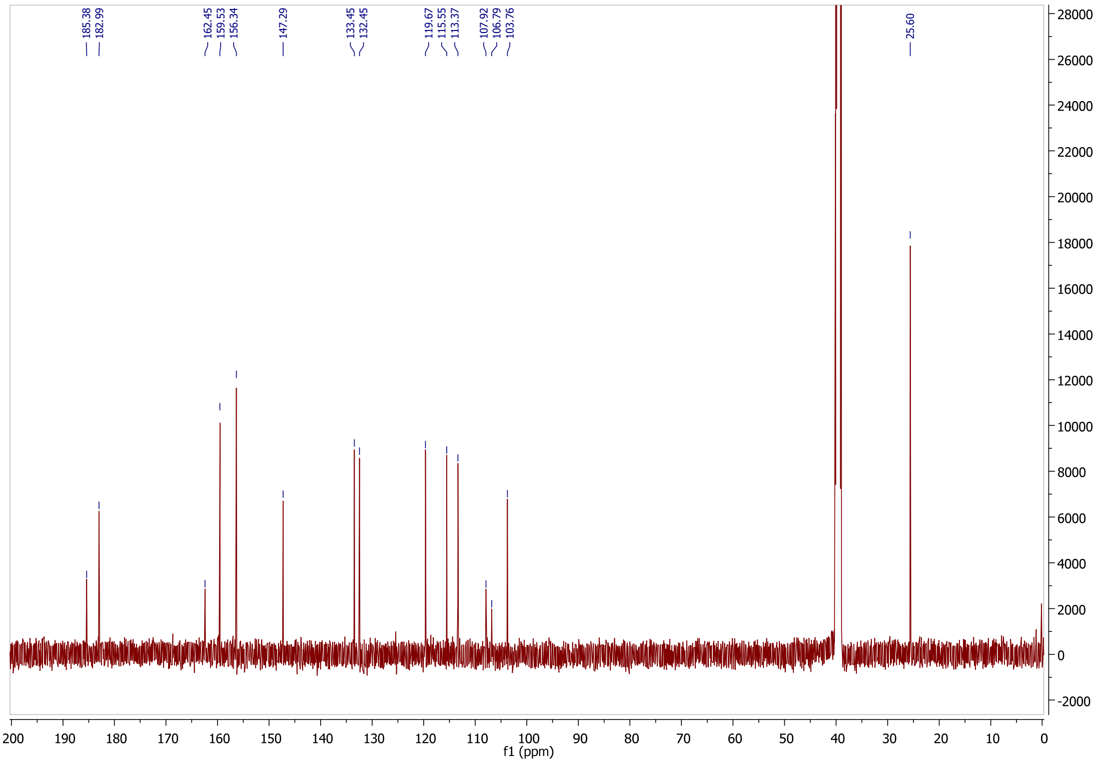

Figure S19. *^{13}C NMR spectrum of compound E_4Br in DMSO, 500 MHz.*

2,4-dichloro-1,3,8-trihydroxy-6-methylanthracene-9,10-dione (E_2Cl).

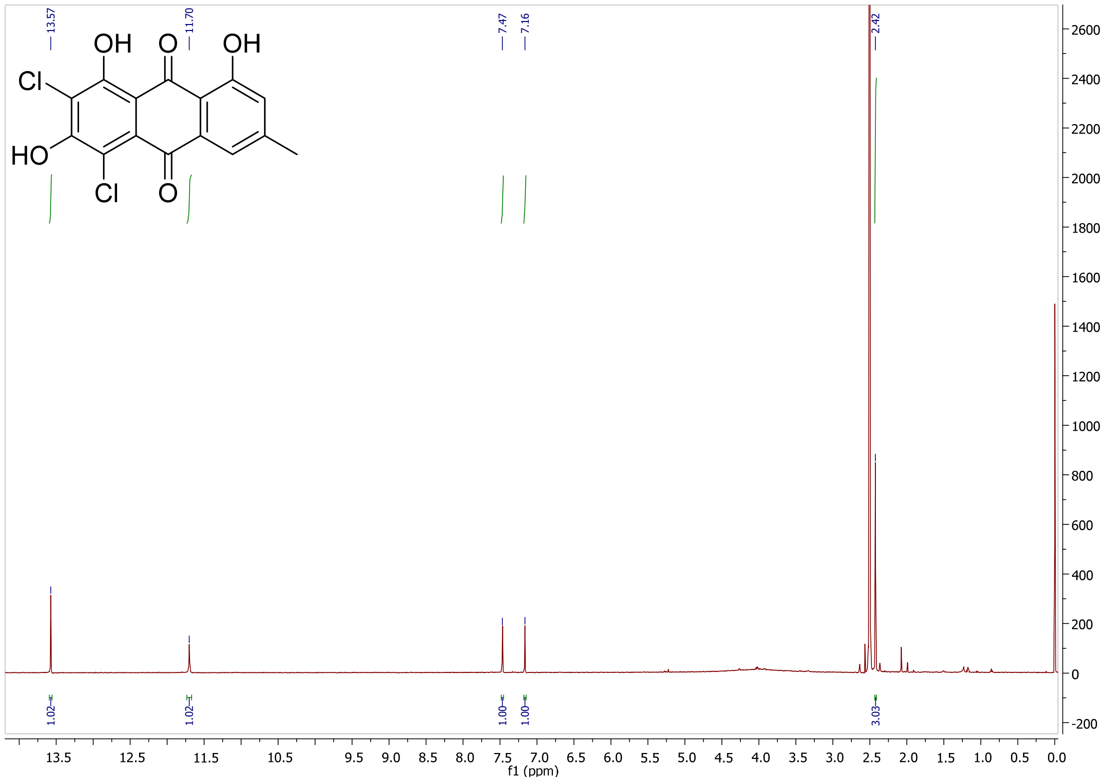

Figure S20. *^{1}H NMR spectrum of compound E_2Cl in DMSO, 500 MHz.*

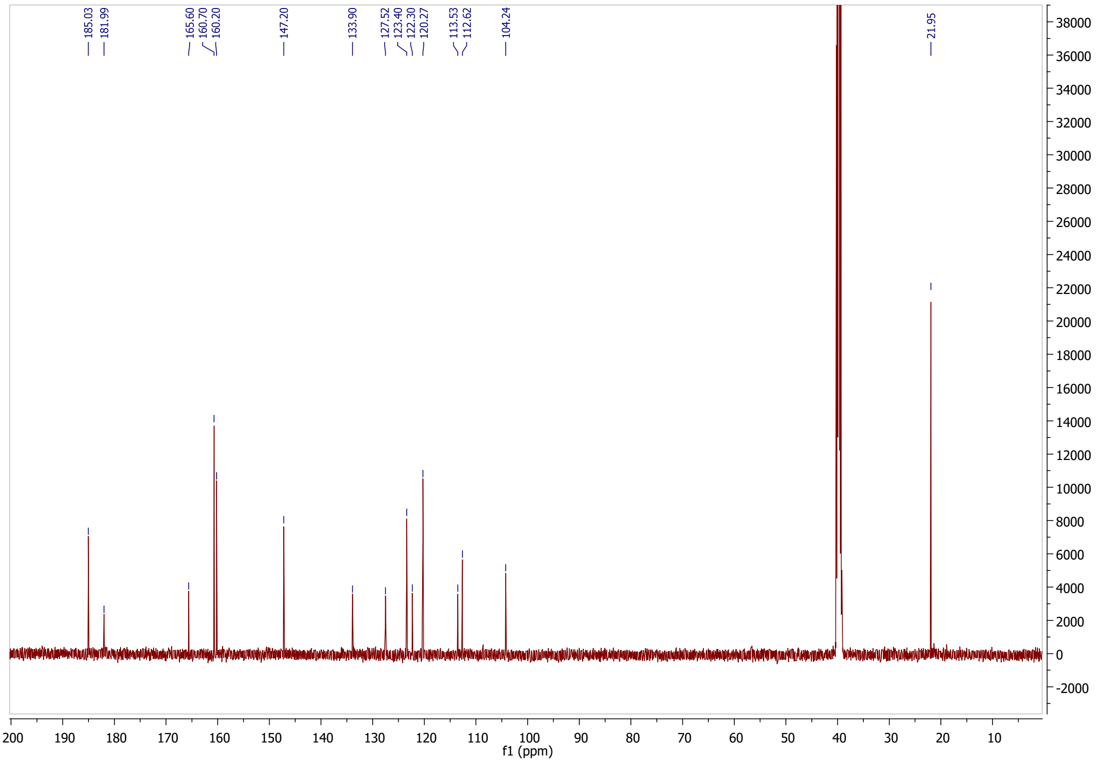

Figure S21. *^{13}C NMR spectrum of compound E_2Cl in DMSO, 500 MHz.*

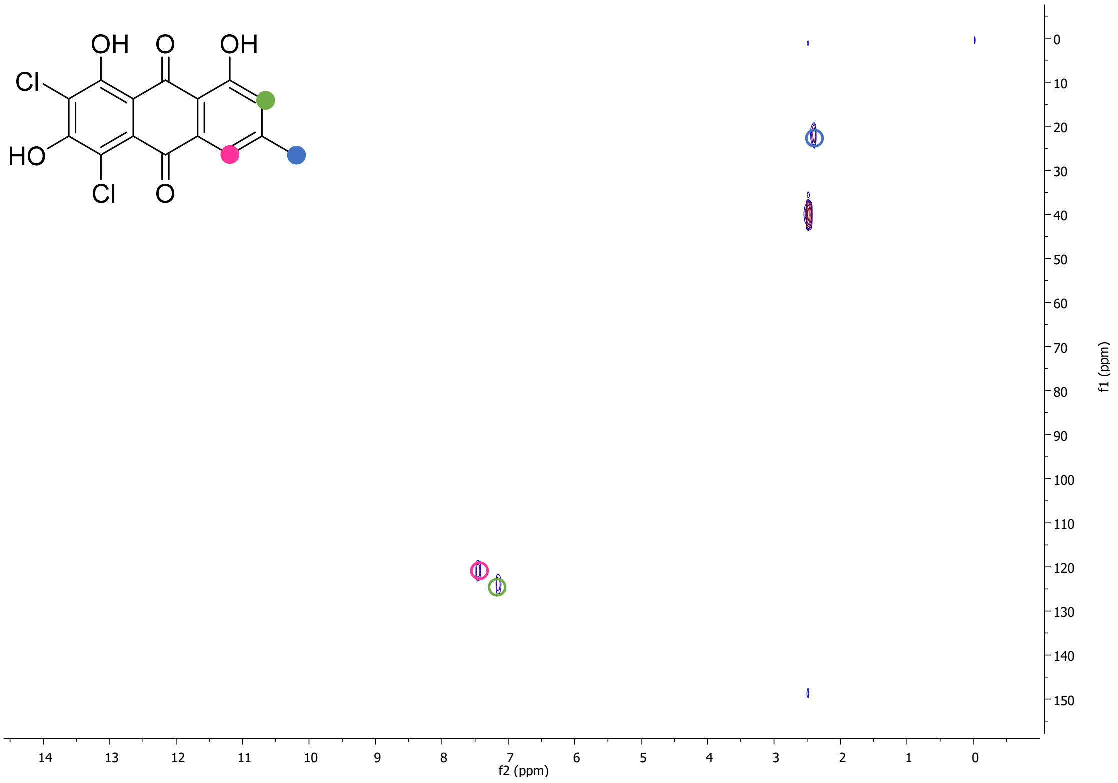

Figure S22. *2D HSQC NMR spectrum of compound E_2Cl in DMSO, 500 MHz.*

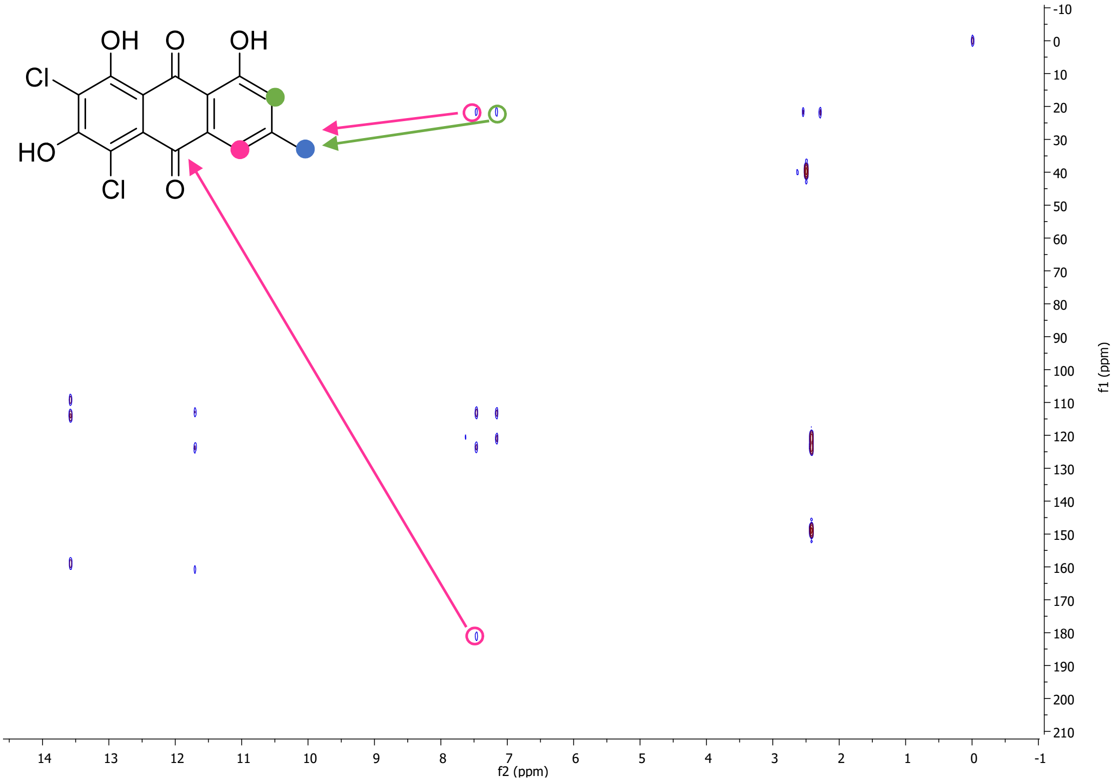

Figure S23. *2D HMBC NMR spectrum of compound E_2Cl in DMSO, 500 MHz.*

1,3,8-trihydroxy-6-methyl-2,4,5,7-tetranitroanthracene-9,10-dione (E_4NO$_2$).

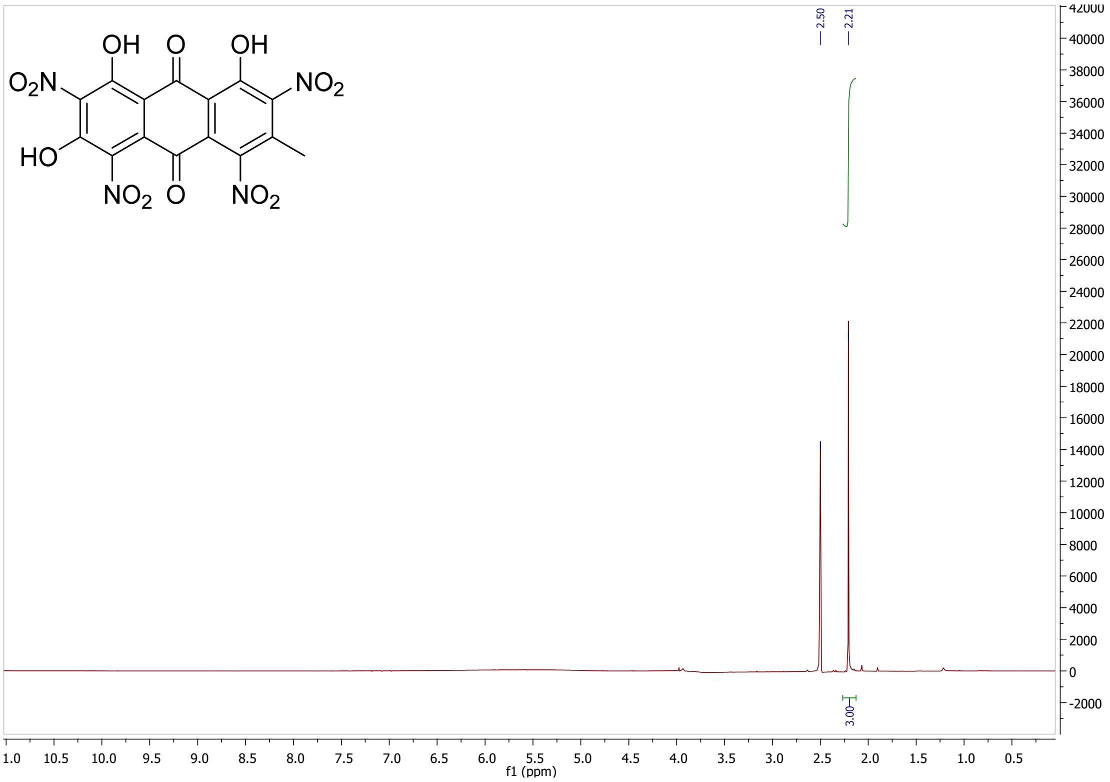

Figure S24. *^{1}H NMR spectrum of compound E_4NO$_2$ in DMSO, 500 MHz.*

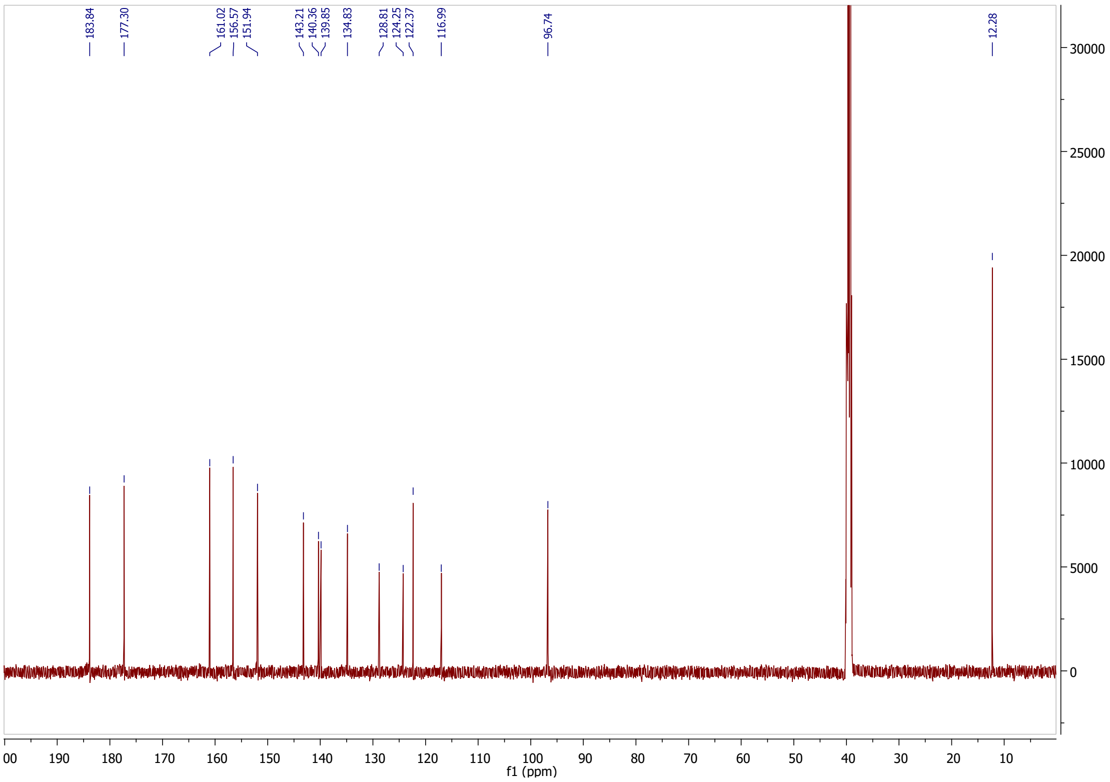

Figure S25. *^{13}C NMR spectrum of compound E_4NO$_2$ in DMSO, 500 MHz.*

4-((3-aminopropyl)amino)-1,3,8-trihydroxy-6-methylanthracene-9,10-dione (E_NH$_2$).

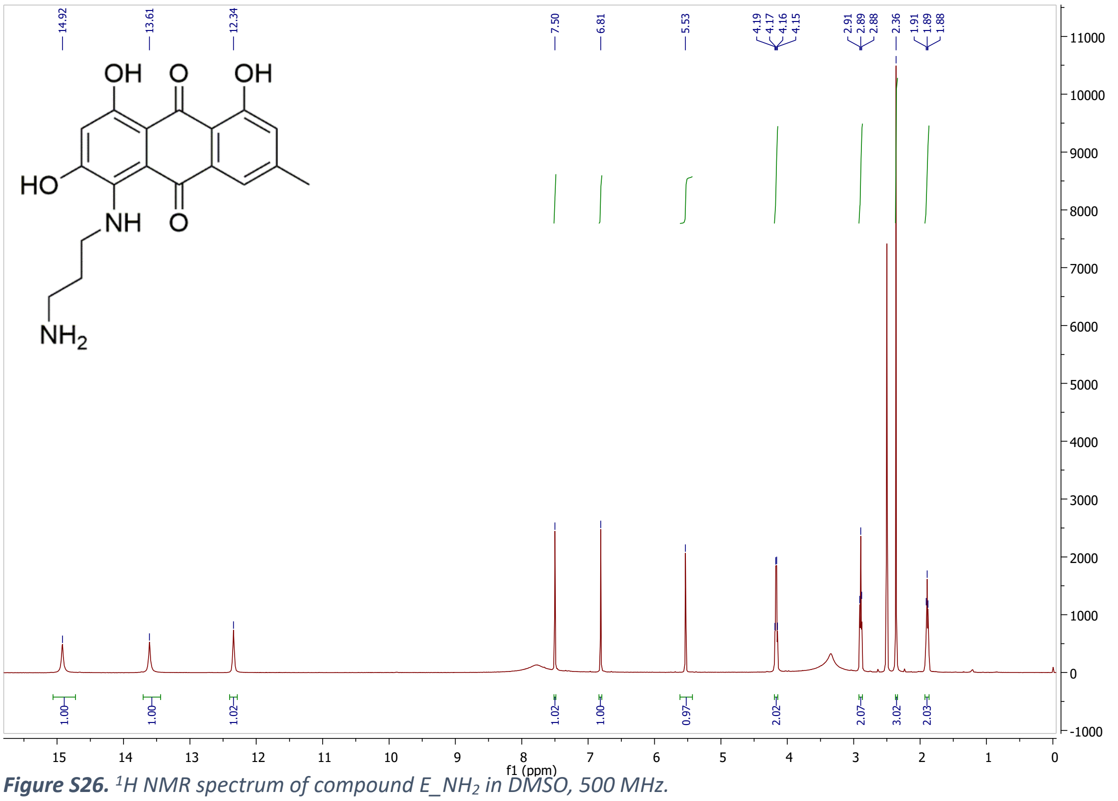

Figure S26. *^{1}H NMR spectrum of compound E_NH$_2$ in DMSO, 500 MHz.*

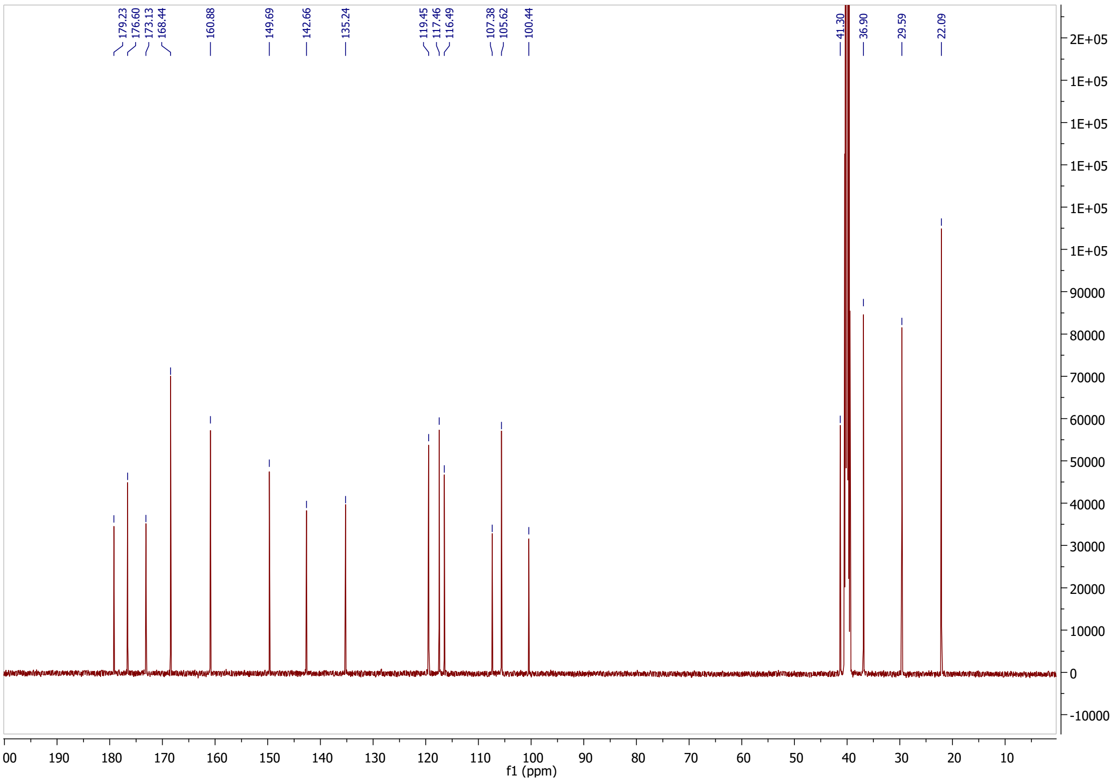

Figure S27. *^{13}C NMR spectrum of compound E_NH$_2$ in DMSO, 500 MHz.*

1,3,8-trihydroxy-6-methyl-9,10-dioxo-9,10-dihydroanthracene-5-sulfonic acid (E_SO_3H).

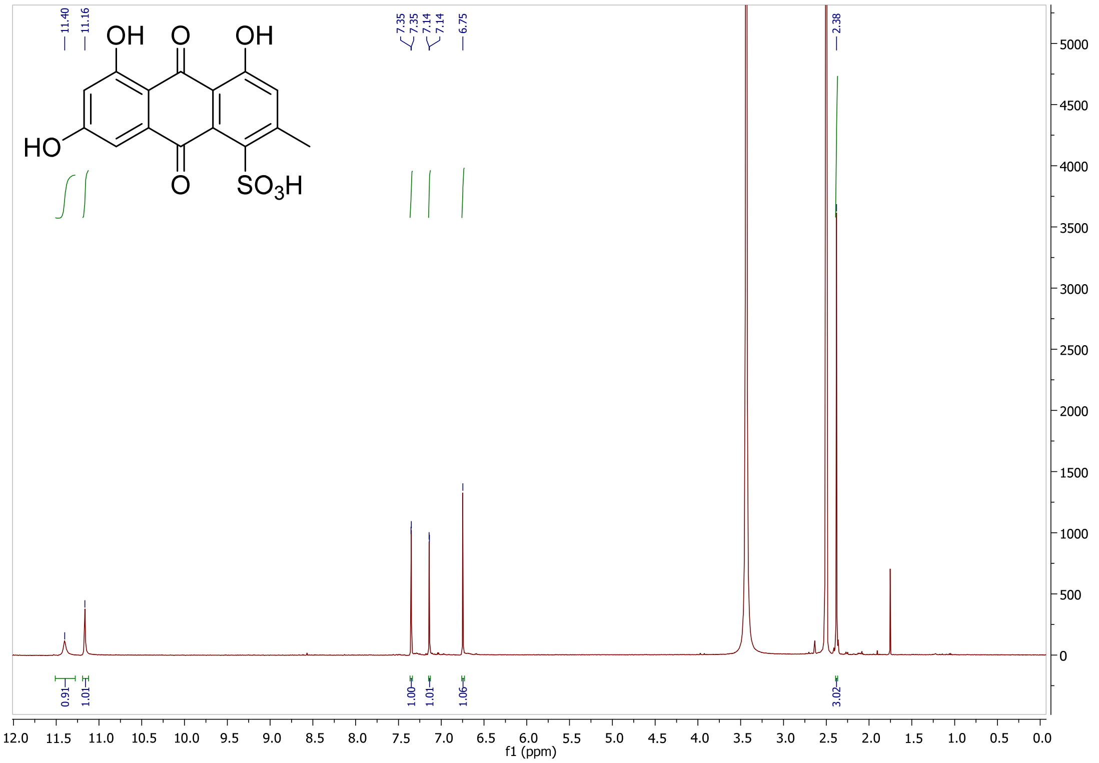

Figure S28. *^{1}H NMR spectrum of compound E_SO_3H in DMSO, 500 MHz.*

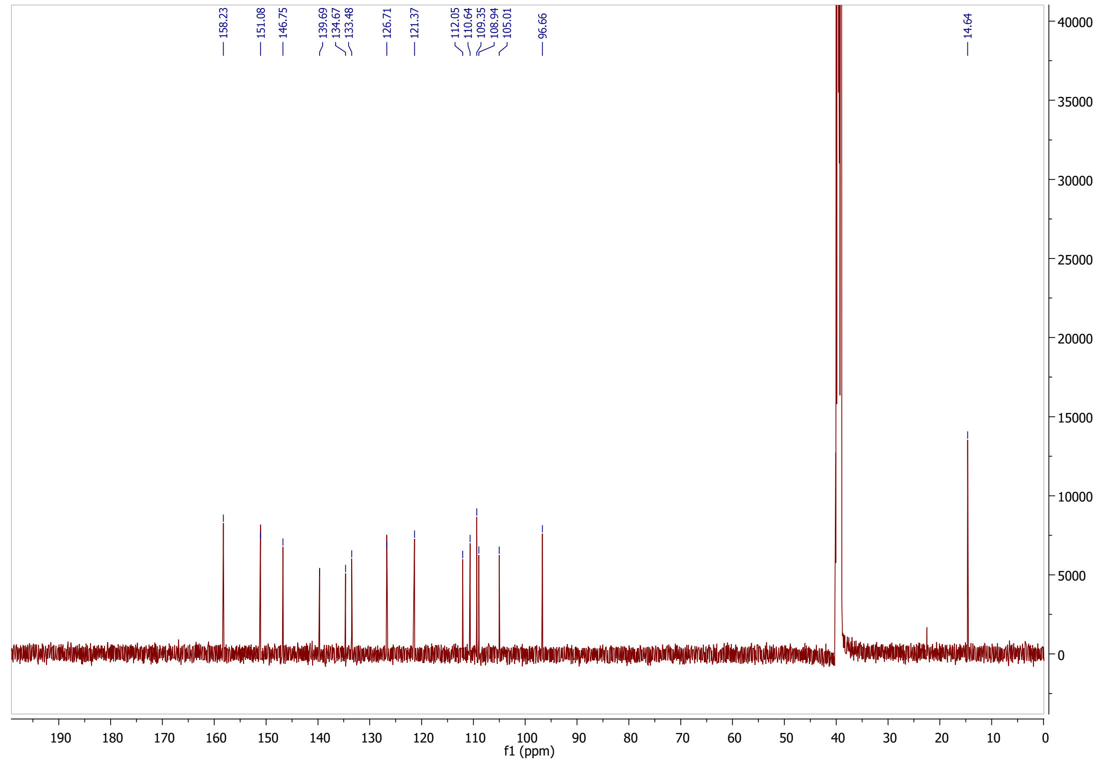

Figure S29. *^{13}C NMR spectrum of compound E_SO_3H in DMSO, 500 MHz.*

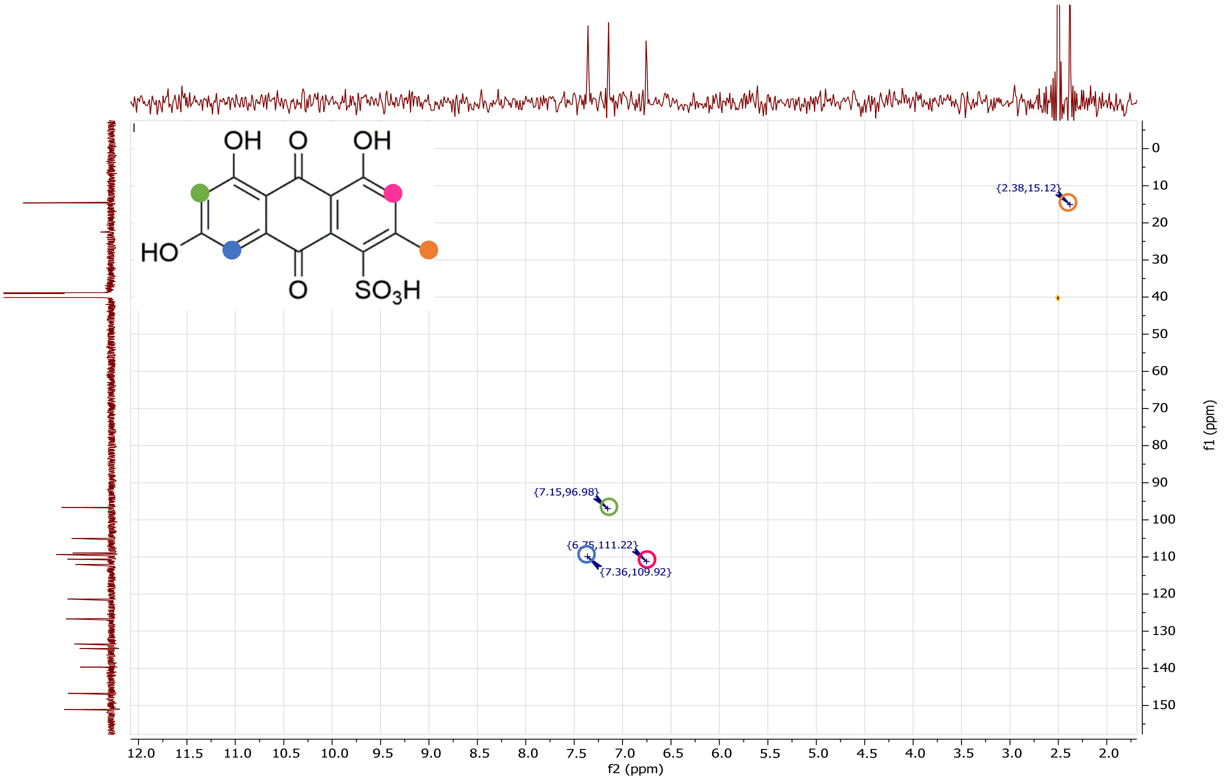

Figure S30. *2D HSQC NMR spectrum of compound E_SO$_3$H in DMSO, 500 MHz.*

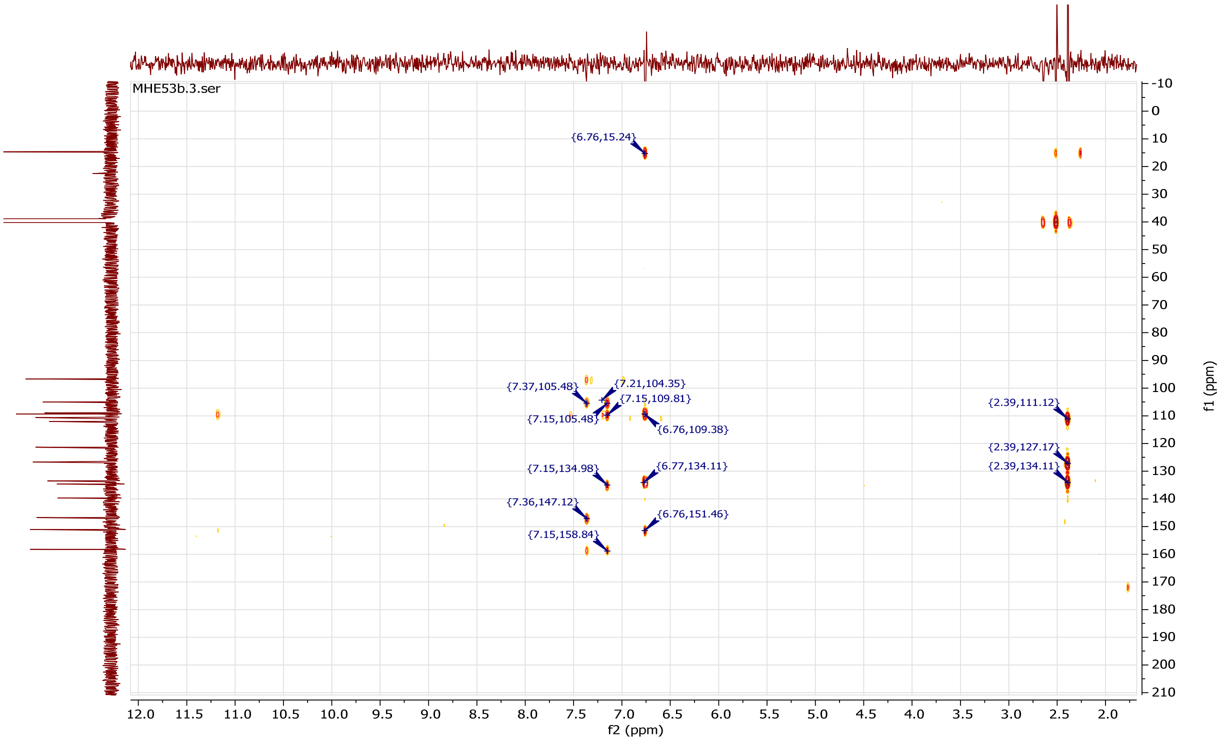

Figure S31. *2D HMBC NMR spectrum of compound E_SO$_3$H in DMSO, 500 MHz.*

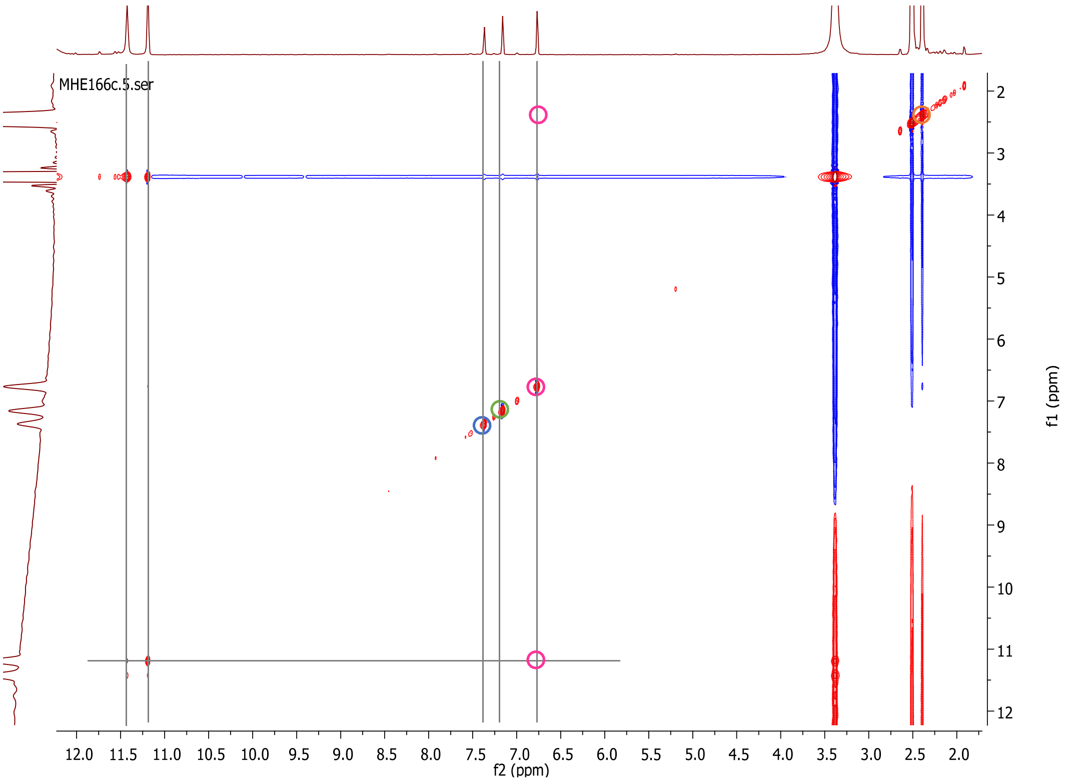

Figure S32. *2D NOESY NMR spectrum of compound E_SO$_3$H in DMSO, 500 MHz.*

1,3,8-trimethoxy-6-methylanthracene-9,10-dione (E_OCH_3).

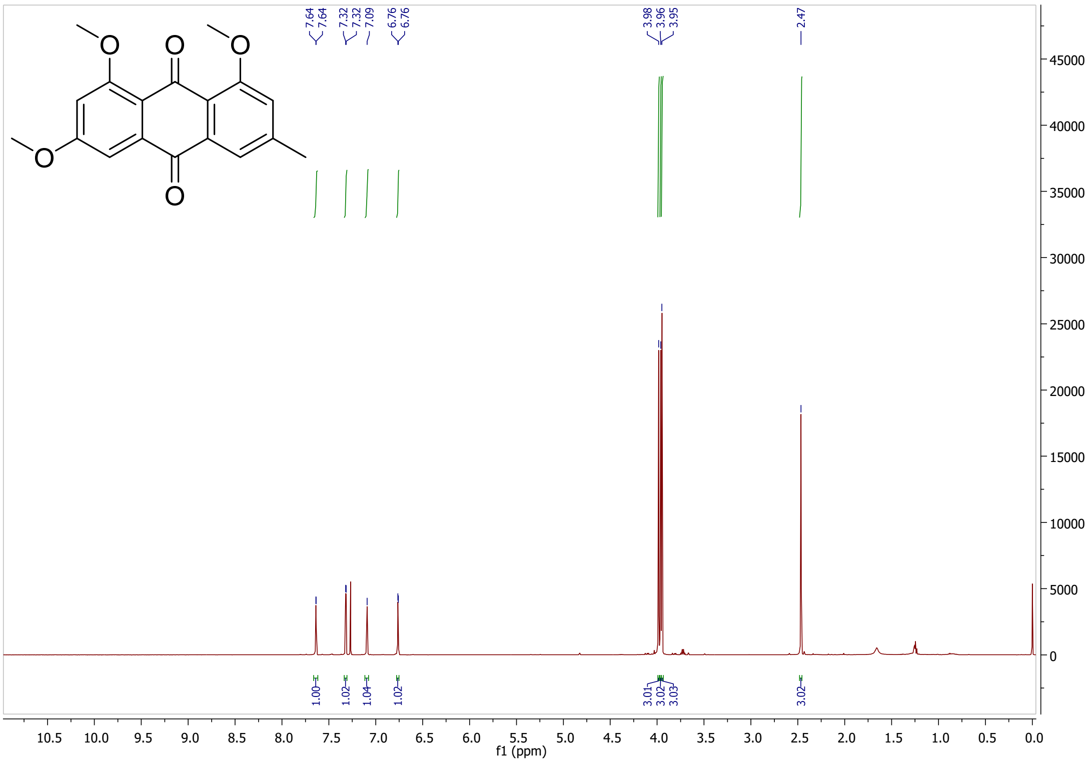

Figure S33. *^{1}H NMR spectrum of compound E_OCH_3 in $CDCl_3$, 500 MHz.*

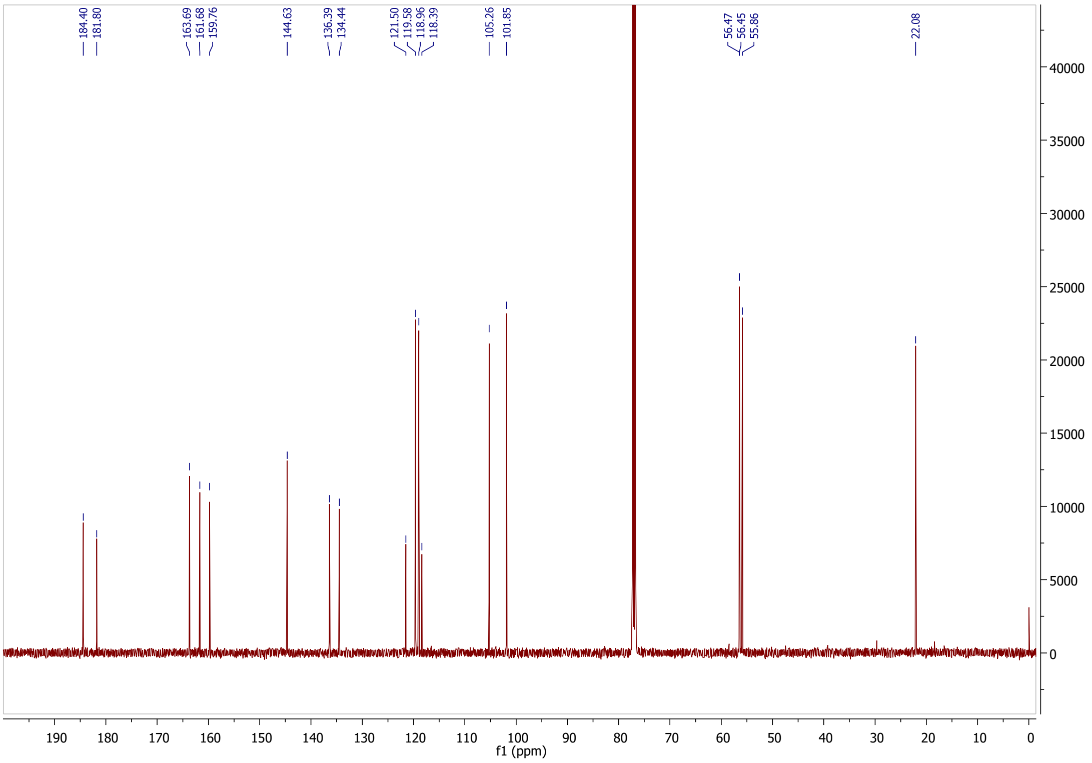

Figure S34. *^{13}C NMR spectrum of compound E_OCH_3 in $CDCl_3$, 500 MHz.*

2,4,5,7-tetrabromo-1,3,8-trimethoxy-6-methylanthracene-9,10-dione (E_Br_OCH$_3$).

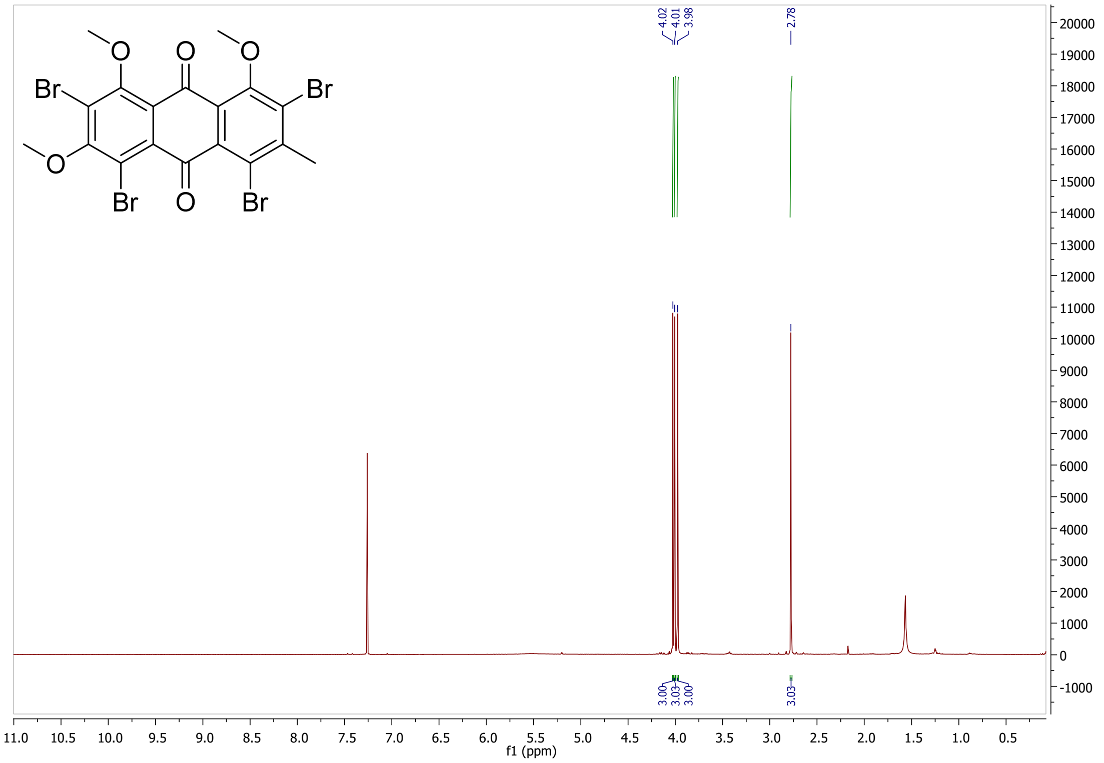

Figure S35. *^{1}H NMR spectrum of compound E_Br_OCH$_3$ in CDCl$_3$, 500 MHz.*

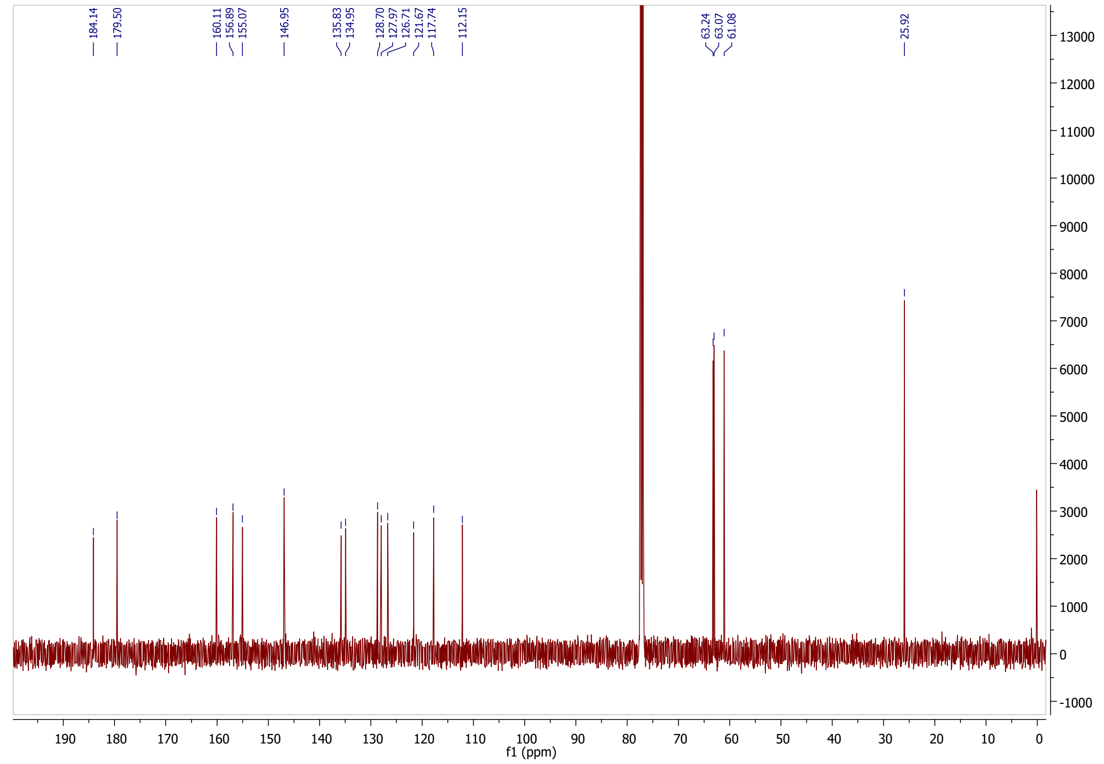

Figure S36. *^{13}C NMR spectrum of compound E_Br_OCH$_3$ in CDCl$_3$, 500 MHz.*

4. LITERATURE

[1] F. Duan, X. Li, S. Cai, G. Xin, Y. Wang, D. Du, S. He, B. Huang, X. Guo, H. Zhao, R. Zhang, L. Ma, Y. Liu, Q. Du, Z. Wei, Z. Xing, Y. Liang, X. Wu, C. Fan, C. Ji, D. Zeng, Q. Chen, Y. He, X. Liu, W. Huang, Haloemodin as Novel Antibacterial Agent Inhibiting DNA Gyrase and Bacterial Topoisomerase I, Journal of Medicinal Chemistry, 57 (2014) 3707-3714.
